# Comprehensive genomic and transcriptomic analyses reveal prognostic stratification for esophageal squamous cell carcinoma

**DOI:** 10.1038/s41392-025-02306-8

**Published:** 2025-07-17

**Authors:** Jian Gao, Qiming Wang, Fangqiu Fu, Yue Zhao, Teng Yang, Xiangze Li, Yihua Sun, Hong Hu, Longfei Ma, Longsheng Miao, Xiaoyang Luo, Ting Ye, Yiliang Zhang, Yang Zhang, Ziling Huang, Hang Li, Longlong Shao, Midie Xu, Kuaile Zhao, Shiyue Zhang, Mou Zhang, Jun Wang, Chong Dai, Xiaoxiao Shang, Tingyi An, Yawei Zhang, Jiaqing Xiang, Zhiwei Cao, Bin Li, Haiquan Chen

**Affiliations:** 1 Department of Thoracic Surgery and State Key Laboratory of Genetics and Development of Complex Phenotypes, Fudan University Shanghai Cancer Center, Shanghai, China; 2 Institute of Thoracic Oncology, Fudan University, Shanghai, China; 3 Department of Oncology, Shanghai Medical College, Fudan University, Shanghai, China; 4 School of Life Sciences, Fudan University, Shanghai, China; 5 Department of Pathology, Fudan University Shanghai Cancer Center, Shanghai, China; 6 Institute of Pathology, Fudan University, Shanghai, China; 7 Department of Radiotherapy, Fudan University Shanghai Cancer Center, Shanghai, China

**Keywords:** Cancer genomics, Tumour heterogeneity, Prognostic markers

## Abstract

Recent large-scale multi-omics studies have characterized the heterogeneity of esophageal squamous cell carcinoma (ESCC), but inconsistent clinical management has hindered the identification of prognostic markers and patient stratification. Here, we conducted genomic and transcriptomic profiling of 203 patients from the ECTOP-2002 study with full clinical information. Mutation in the mucin family, as well as APOBEC signature, were associated with poor prognosis. In contrast, activation of the epithelial-keratinization (EpK) pathway was strongly linked to favorable prognosis and lower post-chemotherapy recurrence rates. Independent validation supported S100A8 + S100A9 complex as a key marker of EpK pathway. Furthermore, we established a prognostic stratification system, FU-ESCC subtyping, which defines three subtypes with distinct molecular and clinical features. The EpK-activated subtype retained characteristics of healthy squamous epithelial cells, showed high expression of the S100A8 + S100A9 complex, and was associated with favorable prognosis. The cancer-associated fibroblast (CAF)-enriched subtype showed elevated FAP and Vimentin expression, abundant CAFs, high proliferative activity, and poor prognosis. The immune-desert subtype was characterized by low immune infiltration, suppressed immune signaling, and similarly poor prognosis. Our study provides a valuable resource and insights to better understand ESCC in the era of precision medicine and targeted therapies.

## Introduction

Esophageal cancer is one of the most lethal malignancies globally, ranking 11th in incidence and 7th in cancer-related mortality, with an estimated 510,716 new cases and 445,129 deaths reported worldwide.^1^ Esophageal squamous cell carcinoma (ESCC) is the predominant histological subtype of esophageal cancer in China,^2^ comprising approximately 85–90% of diagnoses. Despite improvements in surgical techniques, chemotherapy regimens, and perioperative care, the prognosis of ESCC remains dismal. Many patients are diagnosed at an advanced stage and experience locoregional recurrence or distant metastasis even after curative esophagectomy. Furthermore, ESCC displays marked inter- and intratumoral heterogeneity, lacks well-defined molecular subtypes and clear driver mutations, and patients experience highly divergent clinical outcomes. This limitation has hindered the development of effective targeted therapies and suboptimal clinical management.

Recently, several large-scale investigations of ESCC have been conducted, including whole-genome/-exome sequencing (WGS/WES), RNA-seq, proteinomics/phosphor-proteomics and metabolomics, which have revealed the complexity of ESCC.^3–5^ Somatic alterations in *TP53*, *CCND1*, *CDKN2A*, *FAT1*, *NFE2L2*, and *RB1* have been identified as significantly mutated genes.^6^ An international integrative analysis of ESCC revealed substantial heterogeneity in somatic mutation profiles across populations, suggesting population-specific mutational features, particularly among Asian cohorts.^4^ Consistently, a large-scale genomic study involving 1,930 Asian patients identified high-frequency mutations in *TP53*, *MUC16*, *NOTCH1*, *CSMD3*, *KMT2D*, *FAT1*, and *LRP1B*.^7^ Similarly, whole-genome sequencing of Chinese patients revealed a comparable mutational landscape and further demonstrated that *NFE2L2* mutations were significantly associated with poor prognosis.^3^ At the pathway level, mutations in *FAT1*, *FAT2*, *FAT3*, or *FAT4* disrupt the Hippo pathway, while alterations in *NOTCH1*, *NOTCH2*, *NOTCH3*, or *FBXW7* lead to dysregulation of the Notch signaling pathway.^6,8^ Furthermore, mutational signature analysis revealed that Apolipoprotein B mRNA-editing enzyme, catalytic polypeptide-like (APOBEC)-associated signatures were prevalent in most cases and were correlated with tumor stage. In addition, a single-cell spatial transcriptomic map charts the evolutionary trajectory of ESCC, revealing that a proliferative epithelial subpopulation with dedifferentiation and invasive features drives tumor progression. Through JAG1–NOTCH1 signaling, these cells remodel the epithelial–stromal interface, inducing the formation of a CAF–Epi niche that promotes tumor advancement and immune evasion.^9^ Complementing above insights, proteomic and metabolomic analyses have revealed that the immunosuppressive microenvironment cluster in ESCC is characterized by creatine accumulation and a deficiency of hexokinase 3 (HK3), suggesting reprogrammed macrophage metabolism that drives M2-like tumor-associated macrophage polarization.^10^ Moreover, phosphoproteomic profiling uncovered aberrant activation of CDC-like kinase 1 (*CLK1*) signaling in a high-risk ESCC subtype, driven by protein phosphatase 1 (PP1) inhibitors such as CD2BP2 and WBP11. Preclinical models demonstrated that this subtype exhibits therapeutic vulnerability to CLK1 inhibition.^11^ However, the lack of standardized clinical management, long-term follow-up, and comprehensive clinical data in most genomic or transcriptomic studies has limited the development of prognostic analyses, as well as the discovery of reliable biomarkers and prognosis-related patient stratification strategies.

To address this gap, we collected surgically resected specimens from 203 patients enrolled in the ECTOP-2002 randomized clinical trial (Clinical Trial Registration Number: NCT01807936). The ECTOP-2002 study is a prospective, controlled trial comparing esophagectomy with three-field versus two-field lymphadenectomy. This well-annotated cohort demonstrated a five-year overall survival rate of 63%, which was associated only with disease stage, suggesting that it serves as a valuable resource for investigating prognosis-related molecular features. To identify molecular events associated with ESCC prognosis and to establish a stratification system for prognostic risk, we performed WGS and RNA-seq on paired tumor and adjacent non-tumor tissues. Through integrative genomic and transcriptomic analyses, this study aims to uncover survival relevant biomarkers and subtypes that may enhance our understanding of ESCC progression and support the clinical management.

In this study, we performed a comprehensive analysis of the genomic and transcriptomic alterations in ESCC and investigated their associations with clinical prognosis. At the genomic level, we identified the somatic mutation landscape and mutational signatures that are largely consistent with previous studies. Tumor mutation burden (TMB) was not found to be a prognostic factor in patients who had not received neoadjuvant therapy, while lymph node metastasis (LNM) emerged as the most significant factor associated with poor prognosis. Notably, mutations in MUC5B, MUC12, and MUC17 were significantly associated with unfavorable clinical outcomes. At the transcriptomic level, we observed a consistent upregulation of the epithelial-keratinization (EpK) pathway in patients with LNM and advanced clinical stage. Further analysis confirmed that activation of the EpK pathway was significantly associated with favorable prognosis. Moreover, we demonstrated that the S100A8 + S100A9 protein complex serves as a reliable immunofluorescence marker for evaluating EpK pathway activation. Importantly, we proposed a prognosis-related molecular classification system, FU-ESCC subtypes, which stratifies patients into three groups based on distinct molecular features. The EpK-activated subtype is associated with favorable prognosis and a lower recurrence rate after chemotherapy. In contrast, the cancer-associated fibroblast (CAF)-enriched subtype and the immune-desert subtype both exhibit poor prognosis, yet display substantial heterogeneity in tumor microenvironment composition. Together, these findings underscore the molecular heterogeneity of ESCC and offering valuable insights for future personalized treatment approaches.

## Results

### Cohort clinical characteristics of patients

**Fig. 1:**
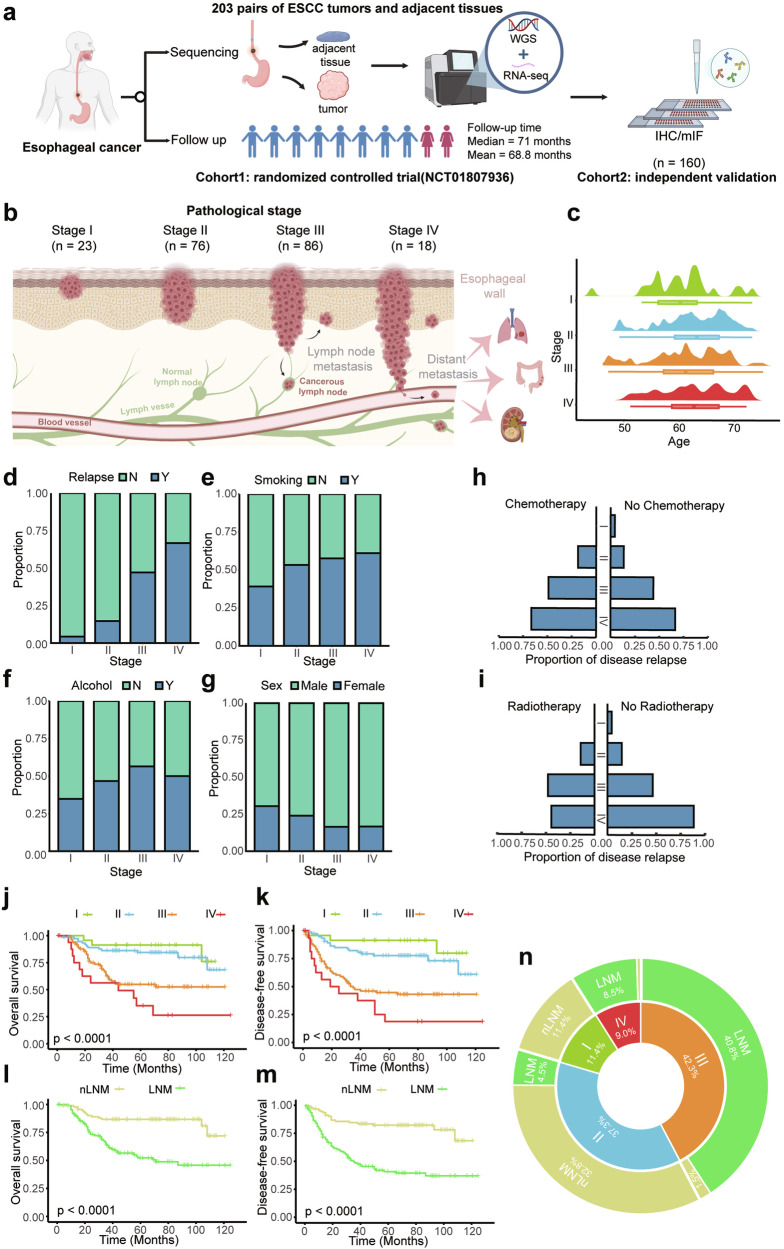
Overall design and clinical characteristics of the study. **a** Schematic representation of the study design. **b** Distribution of clinical staging among ESCC patients. **c**–**g** Age, sex, smoking history, alcohol history and proportion of disease relapse across different pathological stages. **h** Relapse rates in patients who received chemotherapy versus those who did not. **i** Proportion of disease relapse among patients with and without radiotherapy, stratified by pathological stage. **j**, **k** Kaplan–Meier curves for OS and DFS across different pathological stages. **l**–**m** Comparison of OS/DFS between patients with LNM (*n* = 108) and nLNM (*n* = 93). **n** The distribution of LNM status across different pathological stages

The study cohort comprised 203 pairs of treatment-naïve ESCC tumor and adjacent non-tumor tissues collected from the ECTOP-2002 randomized controlled trial (Cohort 1). These samples were obtained from 203 patients with ESCC after esophagectomy with a minimal follow-up of 5 years (mean: 68.7 months; median: 71 months) (Fig. 1a; Supplementary Table 1). WGS and RNA-seq were performed on the paired tissues to enable in-depth molecular profiling (Supplementary Fig. 1a). Additionally, formalin-fixed paraffin-embedded (FFPE) tissue samples from an independent validation cohort (Cohort 2) were prepared for multiplex immunofluorescence (mIF) and other downstream analyses.

The distribution of clinical characteristics for all patients, including pathological stage, age distribution, and tumor location, was displayed in Fig. 1b, c and Supplementary Fig 1b. Most patients were classified as Stage II (36.9%) or Stage III (41.9%), with a median age of 68 years across the cohort (Fig. 1b, c). Advancing pathological stage was associated with higher relapse rates (Fig. 1d) and a greater prevalence of smoking and alcohol history (Fig. 1e, f), along with a slight predominance of male patients in the later stages (Fig. 1g). ESCC was located in the middle third of the thoracic esophagus in 144 of 203 patients (70.9%) and in the lower third in 59 patients (29.1%) (Supplementary Fig. 1b). 41% of patients received chemotherapy and 24% received radiotherapy post-surgery, with these treatments predominantly administered in advanced stages. During follow-up, neither radiotherapy nor chemotherapy significantly affected recurrence rates or overall survival (OS) across II and III stages (Fig. 1h, i; Supplementary Fig. 1c, d). Radiotherapy showed a beneficial impact on OS in stage IV (Fig. 1i; Supplementary Fig. 1d). Among all patients, 5-y OS varied by stage, with Stage I patients exhibiting 5-y OS of 87%, Stage II of 77.8%, Stage III of 60.8%, and Stage IV of 45.8% (Fig. 1j). Similarly, 5-y disease-free survival (DFS) was 82.6% in Stage I, 70% in Stage II, 52.3% in Stage III, and 29.4% in Stage IV (Fig. 1k). Notably, LNM emerged as a critical factor influencing both patient prognosis and recurrence risk (Fig. 1l–n).

### Landscape of somatic mutations in ESCC

**Fig. 2:**
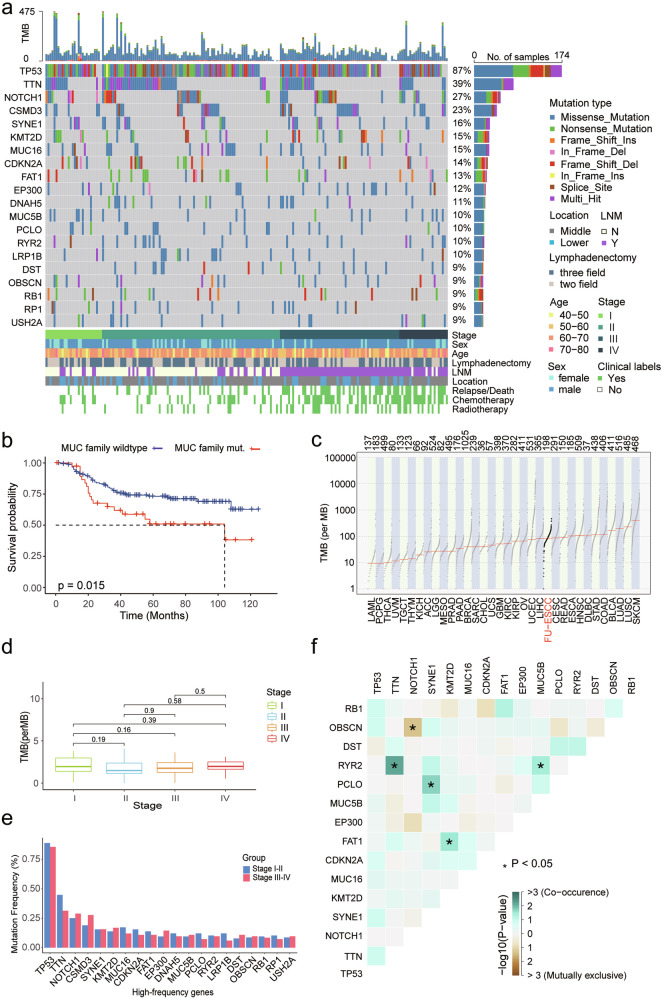
Genomic change in ESCC. **a** Mutation landscape. The top panel displays the TMB for each sample, the middle panel illustrates the distribution of the top 20 most frequently mutated genes across the cohort, and the bottom panel shows various clinical features. Sequencing depth of WGS was 100X. **b** Kaplan–Meier analysis comparing OS probabilities between patients with MUC family wild-type (*n* = 141) and mutated genes (*n* = 58) (*p* value = 0.015). Patients with mutations in at least one of the genes MUC12, MUC17, MUC5B were categorized into the mutation group. **c** Comparison of TMB in ESCC with various cancer types from the Cancer Genome Atlas Program (TCGA). **d** TMB distribution across different pathological stages. **e** Comparison of the mutation frequencies of high-frequency genes across different pathological stages. T-test, *, **, ***, **** indicate *p*-values < 0.05, <0.01, <0.001 and <0.0001, respectively. **f** Co-mutation analysis demonstrating mutual exclusivity and co-occurrence among 15 selected genes. Brown represents mutual exclusivity, while green represents co-occurrence. Asterisks indicate significant co-occurrence

It is generally considered that the progression of cancer is accompanied by an increase in TMB, the accumulation of oncogene and tumor suppressor gene mutations, and structural variants. Therefore, interpreting and comparing the genomic features at each stage is crucial for understanding the molecular changes during disease progression. We first identified somatic mutations and TMB in our cohort. A total of 2,932,597 somatic mutations were detected, comprising 2,758,882 single nucleotide variants (SNVs), 62,794 small insertions, and 110,921 deletions (INDELs) (Supplementary Table 2). The most frequently mutated gene was *TP53* (87%), followed by *TTN* (39%), *NOTCH1* (27%), *CSMD3* (23%), *SYNE1* (16%), *KMT2D* (15%), *MUC16* (15%) and *CDKN2A* (14%) (Fig. 2a). Recapitulating previous reports,^12^ the high-frequency mutated genes identified in our ESCC cohort were largely consistent with those reported in prior studies (Supplementary Fig. 2a).

*TTN* encodes the largest protein in the human body, and mutations in this gene are generally considered functionally irrelevant to tumor biology.^13^ Consequently, there is low selective pressure acting on *TTN* mutations. Additionally, The MUC family genes (*MUC12*, *MUC5B*, *MUC17*) were highly mutated in ESCC, and these mutations were associated with poorer OS compared to the wild-type (Fig. 2b; Supplementary Fig. 2b). Moreover, the mean somatic mutation rate was 2.1 per megabase (Mb) in ESCC (Fig. 2c), with a higher proportion of transversion (Tv) mutations compared to transition (Ti) mutations (Supplementary Fig. 2c). Interestingly, no significant difference in TMB was observed across different pathological stages (Fig. 2d), and TMB levels were not predictive of patient prognosis (Supplementary Fig. 2d).

### Co-mutation status and prognostic relevance

Mutation events in tumors can be mutually exclusive or tend to co-occur, and their co-mutation status has been reported to have prognostic value.^14,15^ In this large single-institution cohort, we conducted a co-mutation analysis focusing on the top 20 most frequently mutated genes with gene expression level (average FPKM > 1) (Fig. 2f, Supplementary Table 3). Specifically, *RYR2* mutations were found to co-occurr with mutations in *TTN* and *MUC5B* (*p*-value < 0.05). Co-occurrence was also observed between *FAT1* and *KMT2D*, as well as between *PCLO* and *SYNE1*. Among all gene pairs analyzed, only *NOTCH1* and *OBSCN* demonstrated statistically significant mutual exclusivity.

We further investigated the prognostic relevance of co-mutated gene pairs in ESCC. Co-mutation of *FAT1* and *KMT2D* was associated with a trend toward favorable prognosis (*p*-value = 0.058) (Supplementary Fig. 2e). However, we found that the co-mutation did not confer a significantly different prognosis compared to *FAT1* mutation alone (Supplementary Fig. 2f).

### Comparison of somatic copy number variations across pathological stages

**Fig. 3:**
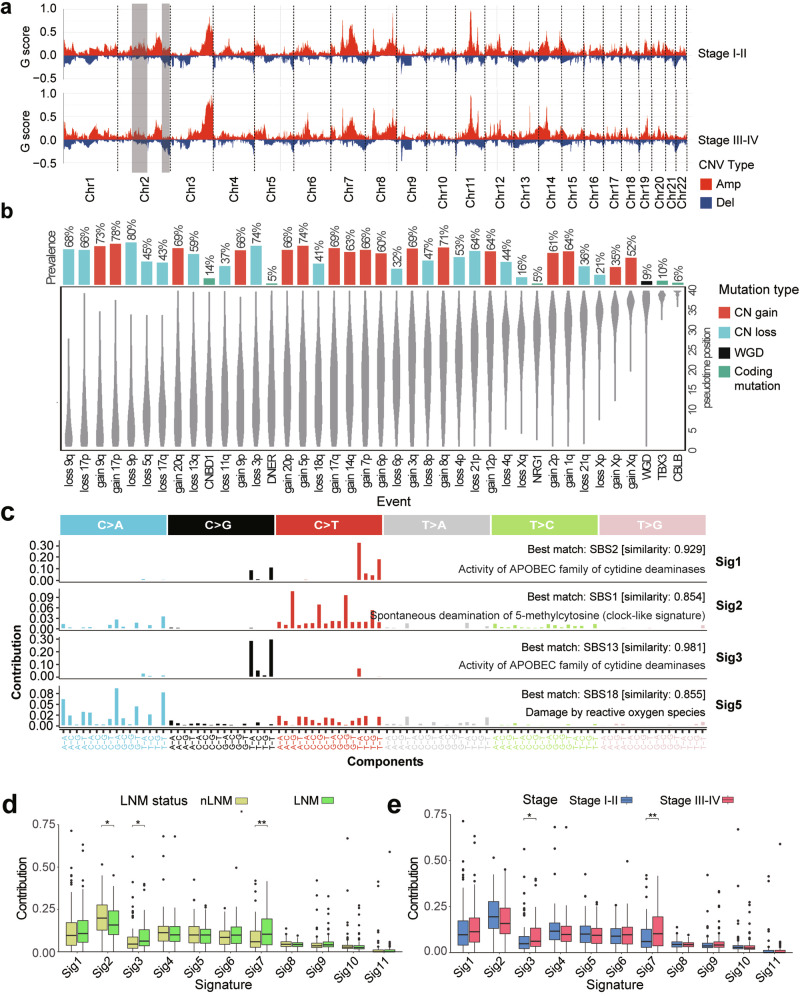
CNV characteristics and mutational signature profiles in ESCC. **a** CNV characteristics across different pathological stages of ESCC. **b** Pseudo-temporal order for genomic events in ESCC. Probability distributions represent the uncertainty in the timing of specific events within the cohort, with event prevalence displayed as a bar plot on the top. A higher event position value indicates that the event occurred later in the pseudo-temporal order by MutationTimeR. **c** Identification of mutation signatures and its similar COSMIC signatures in ESCC. Part of signatures was presented in Extended Data Fig. 3B. **d**, **e** Comparison of ESCC mutational signatures between nLNM and LNM groups, as well as between stages I–II and stages III–IV. T-test, *, **, ***, **** indicate *p*-values < 0.05, <0.01, <0.001 and <0.0001, respectively

Copy number variations (CNVs) were identified and classified as gains or losses across different pathological stages. In Stages I–II, the most frequent CNV event was a gain in chromosome 11q, followed by gains in chromosomes 3q, 7q, 8q, and 14p (Fig. 3a). Similar patterns were observed in Stages III–IV, with additional stage-specific CNV characteristics noted. For example, segments on chromosome 2 transitioned from duplications to deletions as tumor progressed (Stage I–II to stage III–IV) (Fig. 3a). A similar transition was observed when comparing no lymph node metastasis (nLNM) and LNM groups (Supplementary Fig. 3a). The oncogene *MYCN* and the cancer-related gene *NFE2L2* are located on chromosome 2 at 2p24.3 and 2q31.2, respectively. Their gains may contribute to early ESCC tumorigenesis. Notably, previous studies have verified that knockdown of *NFE2L2* promotes the proliferation of ESCC cells,^3^ indicating poorer patient prognosis, which aligns with the chromosome 2 deletions observed in our cohort at stages III–IV.

To elucidate the order of occurrence of genomic changes in ESCC patients, SNVs, indels, and structural variants were assigned to pseudo-temporal order using MutationTiming pipeline.^16^ Compared to other genomic events, losses or gains on chromosomes 9q, 9p, 17p and 11q were among the earliest to occur (Fig. 3b). Furthermore, the frequently mutated ESCC genes *TP53*, *NOTCH1*, *CDKN2A* and *CCND1* are located in these regions. Subsequent events included gains on 7p, 3q, and 8q, where the *EGFR*, *SOX2*, *PIK3CA*, and *MYC* genes are located. Amplification of these genes has been widely reported to improve cancer proliferation, survival, and invasiveness.^14,17–19^ Notably, inhibitors targeting the EGFR pathway (erlotinib and gefitinib) and the PI3K/AKT/mTOR pathway (Alpelisib) have been developed as anti-cancer therapeutics.^20,21^ At last, gains in the 2p region where the proto-oncogene MYCN is located occurred later. MYCN amplification has been widely reported to be associated with poor prognosis in various cancers.^22–24^ Overall, CNV analysis revealed the genomic alterations accompanying ESCC progression and their potential impact on prognosis.

### APOBEC signature correlated with poor clinical outcomes

We investigated mutational signatures in ESCC using an improved non-negative matrix factorization (NMF) algorithm,^25^ identifying 11 mutation signatures (Sig1–Sig11) from our cohort (Fig. 3c; Supplementary Fig. 3b). Among these, Sig4, Sig7, and Sig10 did not correspond to known signatures in the Catalogue of Somatic Mutations in Cancer (COSMIC) database.^26^ The remaining signatures were matched to COSMIC as follows: Sig1 and Sig3 were associated with APOBEC (apolipoprotein B mRNA editing enzyme, catalytic polypeptide-like) activity; Sig2 with spontaneous deamination of 5-methylcytosine; Sig5 with reactive oxygen species (ROS) damage; Sig6 with defective homologous recombination DNA damage repair; and Sig8 and Sig 11 with potential sequencing artifacts. Furthermore, Sig3 (APOBEC signature) activity showed a significant increase in advanced stages (Stage III–IV) and LNM group compared to early stage and nLNM group (Fig. 3d, e), suggesting a potential link to ESCC development and clinical outcomes. Previous studies have demonstrated that APOBEC3B can drive both tumor initiation and evolution in vivo.^27^ Importantly, the association of APOBEC signature activity with metastasis and poor prognosis in ESCC has been previously demonstrated,^3^ aligning with our findings (Supplementary Fig. 3c, d).

### EpK pathway associated with ESCC progression

**Fig. 4:**
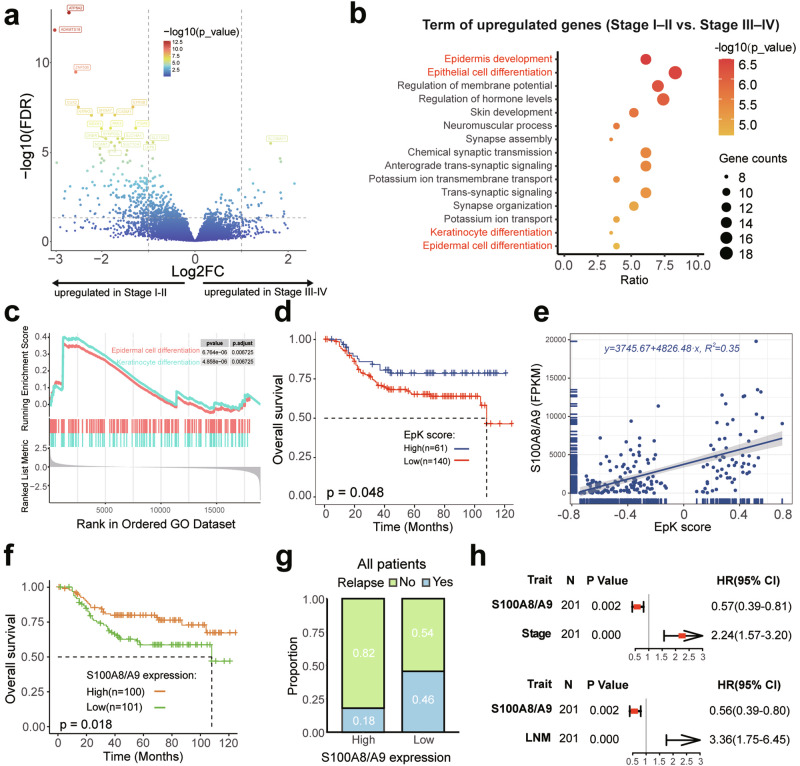
Gene expression alterations of ESCC. **a** Differentially expressed genes (DEGs) between early-stage (Stage I–II) and advanced-stage (Stage III–IV) ESCC. **b** Pathway enrichment analysis of DEGs (Stage I–II vs. Stage III–IV). **c** GSEA enrichment analysis comparing Stage I–II and Stage III–IV. **d** The EpK score distinguishes OS in ESCC. **e** Correlation between S100A8/A9 expression and EpK score, evaluated by linear regression. S100A8/A9 expression was defined as the average FPKM of S100A8 and S100A9. **f** Kaplan–Meier analysis of OS in patients stratified by high and low S100A8/A9 expression levels (grouped by the median). **g** Recurrence rate between high and low S100A8/A9 expression groups in all samples. **h** Multivariate Cox regression analysis for S100A8/A9, clinical stage and LNM status

To further investigate the impact of transcriptional profiling on ESCC clinical outcomes, we systematically analyzed the associations between gene expression and clinical characteristics. A total of 204 upregulated genes and 26 downregulated genes were identified between early-stage (Stage I–II) and advanced-stage (Stage III–IV) ESCC (FDR < 0.05, |log2(FoldChange)| > 1) (Fig. 4a). Further GO (Biological Process) enrichment analysis revealed that genes upregulated in advanced-stage tumors were primarily enriched in pathways such as organic hydroxy compound metabolic process, organophosphate biosynthetic process, and inflammatory response (Supplementary Fig. 4a). In contrast, genes highly expressed in early-stage tumors showed significant enrichment in EpK-related biological processes, including epidermis development, epithelial cell differentiation, keratinocyte differentiation, and epidermal cell differentiation (Fig. 4b, c). Similarly, a comparison between the nLNM and LNM groups revealed consistent findings (Supplementary Fig. 4b, c). These findings strongly suggest a close link between the EpK process and ESCC progression. Subsequently, we curated a gene set representing the EpK pathway (see “Method”) and utilized gene set variation analysis (GSVA) to compute the EpK activity score for each sample. Further prognostic analysis demonstrated that patients with higher EpK score had longer OS time (*p*_value = 0.048) and DFS time (*p*_value = 0.023) (Fig. 4d; Supplementary Fig. 4d).

### S100A8/A9 was an independent prognostic marker

The EpK score requires transcriptomic data for calculation, which limits its applicability for clinical screening and diagnostic support. To address this, simpler markers representative of the EpK pathway need to be identified. Further investigation revealed that *S100A8* (Calgranulin A) and *S100A9* (Calgranulin B) are correlated with the expression of the EpK pathway (Fig. 4e). A single feature, S100A8/A9 (the average expression of *S100A8* and *S100A9*), could significantly distinguish OS and DFS of ESCC (Fig. 4f; Supplementary Fig. 4e). Additionally, ESCC patients with high S100A8/A9 expression exhibited lower recurrence rates (18% vs. 46%) and lower post-chemotherapy recurrence rates (29% vs. 58%) compared to the low-expression group (Fig. 4g; Supplementary Fig. 4f), highlighting its potential as a marker to guide postoperative chemotherapy strategies in ESCC. Importantly, multivariate Cox proportional hazards regression models support that, compared to LNM status and clinical staging, S100A8/A9 serve as independent prognostic factors in ESCC (Fig. 4h). In summary, the EpK pathway was closely linked to ESCC progression and prognosis, with S100A8/A9 serving as a promising predictor for clinical outcomes.

### Prognostic stratification for ESCC

Patient stratification is essential for advancing precision medicine in ESCC. In this study, we explored two distinct strategies to construct prognostic subtypes for ESCC: integrated genomic and transcriptomic profiling, and transcriptomic profiling alone.

We first applied the Cluster-of-Clusters Analysis^28^ (COCA) method to integrate genomic and transcriptomic data for clustering (referred to as GT clustering) (Supplementary Fig. 5a). While the resulting GT clusters showed significant differences in prognosis (*p*-value < 0.001) (Supplementary Fig. 5b), we observed that genomic alterations contributed only marginally to subgroup distinctions (Supplementary Fig. 5a). Moreover, multivariate Cox regression analysis indicated that this clustering result was not independent of LNM status or T stage (Supplementary Fig. 5c).

**Fig. 5:**
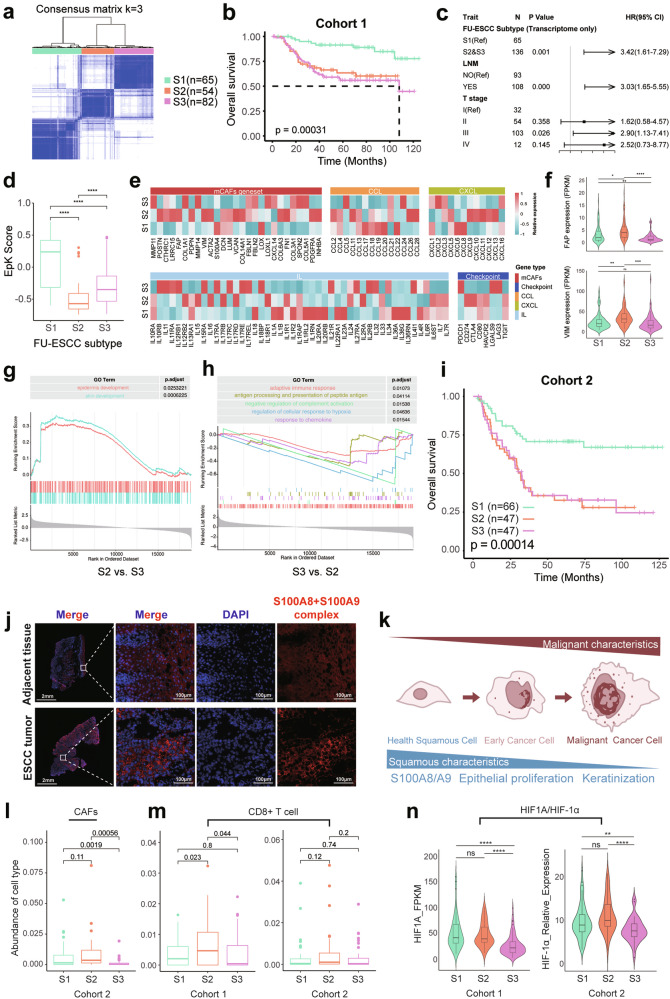
Molecular classification of ESCC. **a** Consensus clustering of Cohort 1 based on transcriptomic profiling. **b** Kaplan–Meier survival analysis showing OS probabilities across the three subtypes in Cohort 1. **c** Multivariate Cox regression analysis incorporating FU-ESCC subtypes, clinical stage, and LNM status in Cohort 1. **d** Comparison of EpK scores across the three subtypes by GSVA in Cohort 1. **e** Expression comparison of mCAF-related genes and immune-related genes among subtypes, including checkpoint genes, chemokines, and cytokines in Cohort 1. Expression values were normalized to a range of −1 to 1. **f** Expression levels of FAP and VIM across the three subtypes in Cohort 1. **g**, **h** GSEA comparing enrichment pathways between S2 and S3. **i** Kaplan–Meier survival analysis showing OS probabilities across the three subtypes in Cohort 2. **j** Subcellular localization of the S100A8/A9 complex in fresh paired ESCC and adjacent normal tissues (*n* = 15, samples independent of Cohort 1 and 2; based on frozen tissue sections). **k** Schematic representation of the potential progression of ESCC cells from healthy squamous epithelium to malignancy. **l**, **m** Comparison of distinct cellular compositions among two cohorts. CAFs were identified as α-SMA + , FAP + , and Vimentin + ; CD8 + T cells were identified as CD3+ and CD8 + . **n** Expression comparison of HIF1A/HIF-1α among two cohorts. *T-test; *, **, **, and **** indicate *p*-values < 0.05, < 0.01, < 0.001, and < 0.0001, respectively

Therefore, we opted to stratify patients using transcriptomic data alone. Through consensus clustering, we identified a three-subtype classification for ESCC, which we named FU-ESCC subtypes (Fig. 5a). This subtyping showed significant prognostic differences (*p*-value < 0.001, Fig. 5b) and was found to be independent of both LNM and T stage (Fig. 5c). Specifically, patients in Subtype 1 (S1) exhibited significantly better OS and DFS compared to Subtype 2 (S2) and Subtype 3 (S3) (Fig. 5b; Supplementary Fig. 5d). The associations between each subtype and mutation profiles, as well as clinical characteristics, are shown in Supplementary Fig. 5e.

To investigate whether the three subtypes exhibited distinct genomic alterations, we analyzed and compared the somatic mutation frequencies. Among the top 100 most frequently mutated genes, only seven showed significant differences in mutation frequency across subtypes (p-value < 0.05, Supplementary Table 4). Specifically, *FAT1* and *HMCN1* were more frequently mutated in Subtype 1, whereas *RYR2*, *ZNF750* and *DMD* showed a lower mutation frequency in this subtype. *FSIP2* mutations were less common in Subtype 2, while *TP53* and *ZNF750* were more frequently mutated in Subtype 3 compared to the other groups.

Interestingly, compared to S2/S3, S1 exhibited higher expression of EpK pathway (Fig. 5d), along with lower LNM and recurrence rates (Supplementary Fig. 6a, b), consistent with previous findings (Fig. 4d, f). Moreover, the majority of patients in S1 demonstrated high expression of S100A8/A9 (Supplementary Fig. 6c). Therefore, we hypothesize that S100A8/A9 can serve as a representative marker for the S1 subtype while also predicting ESCC prognosis.

To further investigate the differences in cellular composition between S2 and S3, we employed multiple deconvolution algorithms (CIBERSORT,^29^ Robust Partial Correlations,^30^ DeconRNASeq^31^ and FARDEEP^32^) and EcoTyper^33^ to predict cellular composition, cell states, and cellular communities based on bulk gene expression data. The cellular composition prediction revealed that, compared to S1, both S2 and S3 exhibited a higher proportion of fibroblasts but a lower abundance of epithelial cells (Supplementary Fig. 6d, e). In addition, EcoTyper identified 10 distinct cell ecotypes and 9 cell states across adjacent non-tumor and tumor samples (Supplementary Fig. 6f). Comparative analysis revealed that CE6 was the dominant cellular ecotypes in adjacent normal tissues, whereas CE1 was predominant across all three tumor subtypes (Supplementary Fig. 6g). Despite the overall similarity in cellular composition between CE6 and CE1 (Supplementary Fig. 6h), they differed substantially in cell states. CE1 was more prevalent in S2, while S3 exhibited a higher proportion of CE9. Notably, CE1 was enriched with fibroblasts and endothelial cells, whereas CE9 primarily consisted of CD4^+^ T cells, CD8^+^ T cells, and B cells. Despite the observed differences in cell ecotype distribution among the subtypes, none of the ecotypes demonstrated a significant correlation with patient survival (Supplementary Fig. 6i). Taken together with the results from the cellular abundance predictions (Supplementary Fig. 6e), these findings suggest that the dominant differential cell populations in S2 are fibroblasts, while in S3 the differences are mainly lymphocytes.

To further examine the functional implications of these compositional differences, we analyzed the expression of CAF gene signatures,^34^ as well as immune-related chemokines, cytokines, and immune checkpoint molecules across subtypes (Fig. 5e). The results revealed that S2 showed significantly elevated expression of CAF-related genes (Fig. 5e, f). Gene set enrichment analysis (GSEA) indicated that pathways related to skin and epidermis development, where fibroblasts exert important functional roles, were significantly enriched in S2 compared to S3 (Fig. 5g). Based on these results, we proposed that S2 represents a CAF-associated subtype, with high expression of *FAP* and *VIM* as potential markers.

Compared to S2, S3 displayed a general trend of lower expression in immune-related gene sets (Fig. 5e). Although genes such as PD1 and LAG3 showed relatively higher expression in S3 compared to S1 and S2, the differences did not reach statistical significance. Moreover, multiple immune response-associated pathways were significantly downregulated in S3 relative to S2 (Fig. 5h), further supporting the notion that S3 may be characterized by suppressed immune activation.

### Validation of FU-ESCC subtypes

To validate above subtype specific characteristic and prognosis relevant, we collected an independent cohort of 160 treatment-naïve ESCC patients (Cohort 2). Tissue microarrays (TMA) were constructed, and three immunohistochemistry (IHC) and multiplex immunofluorescence (mIF) panels were performed. Panel 1 targeted the S100A8 + S100A9 complex (hereafter S100A8/A9 complex) (Supplementary Fig. 7b). Panel 2 included α-SMA, Vimentin, FAP and HIF-1α (Supplementary Fig. 7c). Panel 3 assessed CD3, CD4, CD8, and Foxp3 (Supplementary Fig. 7d). Based on the markers identified in Cohort 1 (S100A8/A9 complex for S1; FAP and Vimentin for S2), we stratified patients in Cohort 2 into the FU-ESCC subtypes. Kaplan–Meier survival analysis revealed that the prognostic trends of FU-ESCC subtypes in Cohort 2 were consistent with those observed in Cohort 1 (Fig. 5i; Supplementary Fig. 7a; Fig. 5b), confirming the prognostic relevance and reproducibility of the FU-ESCC classification.

Patients with high S100A8/A9 complex level (S1) featuring with significantly better prognosis (Fig. 5i; Supplementary Fig. 7a). S100A8 and S100A9 are calcium-binding proteins that form a common heterodimer structure, S100A8/A9 complex. In the immune system, the monomeric of S100A8 and S100A9 protein are primarily expressed in neutrophils and monocytes, playing critical roles in modulating inflammatory responses and inflammation-associated diseases.^35,36^ Conversely, in squamous cell carcinomas, the monomeric of S100A8 and S100A9 protein exhibit an opposite prognostic association.^37–39^ In this study, we found that S100A8/A9 complex are predominantly expressed by squamous epithelium derived cancer cell (Supplementary Fig. 7a). Compared to cancer tissues, RNA expression level of S100A8/A9 was higher in adjacent non-tumor tissues (Supplementary Fig. 7e). Single-cell data analysis further confirmed that S100A8/A9 genes was mainly expressed in healthy squamous epithelial cells (Supplementary Fig. 7f). To investigate the transcriptional regulatory roles of S100A8/A9 complex, we firstly examined the subcellular localization of S100A8/A9 complex in ESCC. Both IHC on cohort2 (Supplementary Fig. 7b) and newly collected ESCC tumor tissues (*n* = 15) showed that the S100A8/A9 complex was localized in the cytoplasm rather than the nucleus (Fig. 5j, down panel). Similarly, in normal esophageal squamous epithelial cells, S100A8/A9 complex was also localized in the cytoplasm (Fig. 5j, up panel). Based on these observations, we infer that S100A8/A9 complex is unlikely to function as transcriptional regulators in ESCC. In addition, we explored the potential of the S100A8/A9 complex as a non-invasive biomarker. However, no significant correlation was observed between the levels of S100A8/A9 complex in serum and tumor tissues (Supplementary Fig. 7g), suggesting that circulating S100A8/A9 complex level may not serve as a reliable predictor of patient prognosis. Overall, our evidence suggested that high expression of the EpK pathway indicates that cancer cells retain more squamous epithelial characteristics rather than malignant features, which was associated with better patient prognosis (Fig. 5k). S100A8/A9 complex serves as effective surrogate marker to detect activation of epithelial-keratinization pathway in ESCC.

S2 was validated as a CAF-enriched subtype, with mIF confirming its higher abundance of CAFs (Fig. 5l). Previous studies have reported that the transformation of fibroblasts into CAFs promotes tumor progression in ESCC.^40^ The features of S2 suggest that patients within this subtype may require closer clinical monitoring and could potentially benefit from clinical trials targeting CAFs. In addition, S2 displayed increased immune cell infiltration (Fig. 5m; Supplementary Fig. 7i–j) and elevated expression of proliferation-associated markers, including Proliferating Cell Nuclear Antigen (*PCNA*), Hypoxia-Inducible Factor 1 A (*HIF1A*), and Aurora Kinase A (*AURKA*) (Supplementary Fig. 7k). These findings indicate that S2 is characterized by both CAF enrichment and active cellular proliferation, features that may contribute to poor prognosis and potential therapeutic resistance.

In the validation cohort (Cohort 2), S3 was defined as patients who did not satisfy the classification criteria for either S1 or S2. This subtype was characterized by reduced immune cell infiltration and lower activity of immune-related pathways (Fig. 5m; Supplementary Fig. 7i–j; Fig. 5h). In this study, we interpret S3 as an immune-desert-like subtype, a feature that may imply limited responsiveness to current therapeutic strategies.

**Fig. 6:**
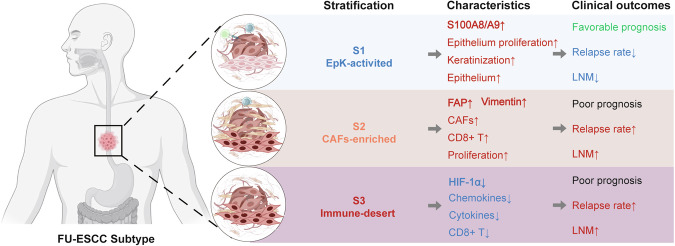
Prognostic stratification of ESCC. Schematic representations of the tumor microenvironment for each subtype are shown within the circles. The key characteristics of each subtype, including gene markers, biological pathways, and cellular subtypes, are listed respectively. Red upward arrows indicate higher levels of a feature in the corresponding subtype, while blue downward arrows indicate lower levels

In summary, by integrating molecular markers, cellular composition, and clinical features, we established three prognostic ESCC subtypes, termed FU-ESCC Subtypes (Fig. 6). The EpK-activated subtype (S1) represents tumors with transcriptomic profile resembling healthy squamous epithelial cells, exhibiting the best clinical outcomes among the three subtypes. The CAF-enriched subtype (S2) is characterized by high fibroblast abundance, elevated expression of CAF-related and proliferation markers, and is associated with poor prognosis and potential chemotherapy resistance. The Immune-desert subtype (S3) displays low multiple immunosuppressive features, correlating with poor prognosis. FU-ESCC stratification provides a valuable insight for clinical decision-making, guiding postoperative management, and informing the development of personalized treatments for ESCC.

## Discussion

In this study, we comprehensively characterized the genomic and transcriptomic landscape of ESCC by performing WGS and RNA-seq on paired tumor tissue and adjacent noncancerous specimens from 203 patients enrolled in a rigorously designed randomized clinical trial (NCT01807936). Different to previous studies, our explored cohort benefits from standardized surgical and perioperative protocols, complete clinical annotations, and extensive long-term follow-up, enabling robust prognostic analyses free from confounding effects of neoadjuvant therapy. The scientific community recognizes that systemic chemotherapy can induce significant genomic and transcriptomic alterations in tumor tissues, potentially interfering the identification of prognostic features. Leveraging this high-quality dataset, we systematically identified prognostic genomic alterations and transcriptomic signatures and established the FU-ESCC molecular subtypes, which correlate with distinct clinical outcomes and tumor microenvironment features. These integrative analyses provide critical insights into ESCC heterogeneity and offer promising avenues for improving patient stratification and personalized therapeutic strategies.

*FAT1* mutations have been frequently reported in ESCC, and knockdown or endogenous expression loss of *FAT1* has been shown to accelerate cell migration and invasion.^41^ *KMT2D*, a histone methyltransferase, plays a pivotal role in epigenetic regulation and has been implicated in various cancers as a potential oncogene.^42^ Although our co-mutation analysis revealed that *FAT1*-*KMT2D* co-mutations were associated with a trend toward better prognosis, this survival benefit was not significantly greater than that conferred by *FAT1* mutation alone. Notably, none of the other co-mutated gene pairs showed a statistically significant impact on prognosis. In addition, the MUC family consists of high-molecular-weight glycoproteins, and elevated mucin production is frequently observed in various malignancies. Mechanistically, the inhibition of NOTCH pathway, a pathway frequently mutated in ESCC, was associated with upregulation of glandular mucins (MUC2, MUC3B, MUC5B, MUC17) and downregulation of squamous keratins.^43^ In this study, mutations in MUC family genes were significantly associated with poor prognosis. Although MUC family gene mutations have been reported to be significantly more prevalent in colorectal cancer among Chinese populations compared to Western cohorts,^44^ research on the role of MUC genes in esophageal cancer remains limited. Further studies are needed to elucidate their prognostic mechanisms.

S100A8/A9 has emerged as a multifaceted biomarker in ESCC. Known for its regulatory roles in the immune system and as a marker of neutrophils, S100A8/A9 has demonstrated opposing effects across different cancer types. In adenocarcinomas, its expression is primarily contributed by immune cells and has been closely associated with immune infiltration.^35^ In contrast, in squamous cell carcinomas, S100A8/A9 expression is predominantly derived from epithelial cells, particularly healthy squamous cells. In ESCC, prior studies have linked elevated peripheral blood levels of S100A8 with responsiveness to neoadjuvant chemotherapy.^45^ Moreover, one report showed that forced nuclear localization of S100A8/A9 monomers could promote the transcription of cancer-associated genes and enhance breast cancer cell transformation,^46^ suggesting that the subcellular localization of S100A8/A9 may underlie its context-dependent functions. However, this hypothesis requires further systematic investigation. In our study, we offer a new perspective. By analyzing untreated surgical tissue samples, we found that S100A8/A9 gene expression primarily reflected tumor cell characteristics rather than immune status. Importantly, we further demonstrated that a single antibody targeting the S100A8/A9 complex was sufficient to distinguish a patient subtype with favorable postoperative prognosis, highlighting its potential as a clinically applicable biomarker for patient stratification.

ESCC remains a disease with poor prognosis, and achieving precision treatment remains a formidable challenge. In Cohort 1 of our study, although none of the patients received neoadjuvant therapy, their survival outcomes were better than those previously reported.^40^ Among the three subtypes identified in our study, S1 demonstrates a markedly favorable prognosis, regardless of whether adjuvant therapy is administered. This suggests that surgery alone may be sufficient for patients in this group, allowing them to avoid the side effects associated with adjuvant treatments.

CAF enrichment is a well-recognized indicator of poor prognosis in various cancers.^47^ Recent single-cell spatial transcriptomic analyses have also identified CAF-associated spatial niche as being linked to poor prognosis.^9^ Currently, there are no dedicated clinical trials targeting CAF specifically in ESCC. Encouragingly, promising CAF-targeting therapies have emerged in other solid tumors, including approaches targeting surface markers such as FAP (e.g., OMTX705, an antibody-drug conjugate), secreted factors like TGF-β, and key signaling pathways such as FGFR.^48^ The CAF-enriched ESCC subtype identified in our study may potentially benefit from these innovative targeted therapies in the future.

Immune checkpoint inhibitors (ICIs) have become a focal point in ESCC treatment, with over 20 registered clinical trials. However, response rates to ICIs in ESCC remain low (13–28%).^49^ Patient stratification is critical to identify those most likely to benefit from ICIs. Some clinical trials already utilize recruitment strategies based on specific biomarkers, such as PD-L1 expression, TMB, or immune infiltration status. In our study, S2 exhibited a higher level of immune infiltration, indicating potential responsiveness to immunotherapy and offering implications for advancing precision medicine with ICIs.

There are some limitations in our study. We employed the widely used multi-tool to estimate cellular composition from bulk RNA-seq data. This approach lacked detailed compared to single-cell sequencing, which can provide finer insights into the tumor microenvironment and cell-cell interactions. On the other hand, single-cell sequencing relies on fresh tissue samples, often lacking long-term follow-up. To partially validate our findings from Cohort 1, we performed mIF staining on Cohort 2 using three panels to assess the expression of key markers and cell abundance. Nevertheless, mIF has limited throughput and cannot capture the full complexity of the tumor microenvironment. Moreover, protein degradation over prolonged storage time in tissue microarrays may contribute to inconsistencies in validation experiments, a phenomenon particularly evident in the detection of PD1.^50^ Emerging spatial transcriptomics technologies compatible with FFPE samples may overcome these limitations and offer transformative potential for ESCC research.

In conclusion, our work provided a valuable resource and insights into the molecular underpinnings of ESCC progression and prognosis. By integrating multi-omics data with long-term clinical outcomes, we identified distinct molecular subtypes with potential clinical relevance, including therapeutic vulnerabilities and prognostic biomarkers. These findings not only advance our understanding of ESCC biology but also lay the groundwork for future precision medicine strategies in ESCC management.

## Materials and methods

### Sample collection

This single-center, open-label, randomized trial was conducted in Fudan University Shanghai Cancer Center in Shanghai, People’s Republic of China. The study was registered in ClinicalTrial.gov (NCT01807936). The trial complied with the principles of the Declaration of Helsinki and the guidelines for good clinical practice. The protocol was approved by the Institutional Review Board of Fudan University Shanghai Cancer Center. All enrolled patients provided a written informed consent. The surgeries were performed by 10 thoracic surgeons in the Shanghai Cancer Center (HQC, JQX, YWZ, YHS, HH, HCL, JZ, LSM, LFM, and BL). We gathered a total of 406 surgical specimens (comprising both tumor and adjacent tissue) from 203 treatment-naïve patients from this trial (Cohort 1). Subsequent to performing whole-genome sequencing and transcriptome sequencing for each specimen. We also included 160 treatment-naïve patients diagnosed with ESCC as a validation cohort, and their specimens were prospectively collected, with 2–4 replicate cores per patient. (Cohort 2). Additionally, to assess the correlation between S100A8/A9 protein levels in tumor tissue and blood, we collected 15 paired ESCC tumor samples and corresponding blood specimens.

### DNA extraction and whole-genome sequencing

The acquisition of tissue samples requires coordination and confirmation with the hospital. About 200 ng high-quality genomic DNA was sheared with Covaris LE220 Sonicator (Covaris) to about 350 bp. The library was constructed according to the protocol of TruSeq Nano DNA Sample Prep Kits (Illumina). First the fragmented DNA was purified using sample purification beads, and the product was repaired by the end, and A base was added to the 3 ‘end. Then, the adapters are ligated with the specific barcode sequence. The CleanNGS magnetic beads (CleanNA) were used to screen out incomplete connections and self-connecting products. Sequencing libraries were formed by PCR amplification using universal primers complementary to the adapter sequences. After library construction, Qubit 3.0 fluorometer dsDNA HS Assay (Thermo Fisher Scientific) was used to quantify concentration of the libraries, while the size distribution was analyzed by Agilent Bioanalyzer 4200 (Agilent). Paired-end sequencing was performed using the NovaSeq 6000 S4 Reagent Kit v1.5 (300 cycles) on Illumina NovaSeq 6000 platform (Illumina, San Diego, USA). The average WGS depth of coverage is 50X.

### Somatic mutation calling

High-quality reads were aligned to the UCSC human reference genome (hg38) using Burrows−Wheeler Aligner (BWA v.0.7.17)^51^ with default parameters. Somatic SNVs and InDels were jointly called Mutect2 (v4.1.1.0) ^52^ and Strelka.^53^ Only variants passing both quality filters were included in downstream analyses. Mutations in low complexity regions, such as tandem repeat regions and highly homologous genomic regions, were excluded. Low-confidence variants were discarded if any of the following criteria were not met: total depth > 10, alternative allele depth > 3, and mutation frequency > 0.01. Subsequently, all high-confidence mutations were annotated using ANNOVAR (Version 2019-10-24).^54^

### Mutual exclusivity and mutation co-occurrence analysis

Mutually exclusive or co-occurring sets of genes were detected using the somaticInteractions function in the Maftools R package^55^ using pair-wise Fisher’s exact test (*p* < 0.05) was employed to identify differentially mutated genes between our and literature cohorts.

### Copy number variant analysis

Copy number variations were detected using Control-FreeC (Version 11.6).^56^ GISTIC2 (v2.0.23)^57^ is utilized for analyzing CNV amplification or deletion changes at the arm, focal, and gene levels. Each sample’s copy number variation segment file was obtained using Control-FreeC, and these segment results were employed as input for GISTIC2. In the cohort, significant changes in arm events were identified using a threshold of *q* < 0.01.

### Mutation timing pipeline

#### Step 1: copy number variation detection and data integration

To identify CNVs in tumor samples, we utilized ASCAT (v3.1.2) (https://github.com/VanLoo-lab/ascat). ASCAT was applied to all samples to estimate allele-specific copy number profiles, as well as tumor purity and ploidy. From the ASCAT output, the local_cn values were extracted for downstream analysis. The extracted local_cn data were integrated with existing mutation annotation format (MAF) files and variant call format (VCF) files for each sample. This integration produced a comprehensive dataset that combined somatic mutation information with CNV data, serving as the input for subsequent analyses.

#### Step 2: clustering and timing analysis

The integrated datasets were processed using PhylogicNDT (v1.0) (https://github.com/broadinstitute/PhylogicNDT) to perform clustering and timing analyses.

Clustering: Mutations were clustered based on their cancer cell fraction (CCF), grouping mutations with similar evolutionary patterns. This step allowed the identification of mutation clusters that reflect distinct subclonal populations within the tumor.

Timing: The temporal relationships between mutations and CNV events were inferred using PhylogicNDT’s timing module. This analysis determined the chronological order of mutations relative to CNV events, yielding a mut_ccf_file for each sample. The mut_ccf_file contained mutation clusters, their corresponding CCF values, and timing information.

#### Step 3: phylogenetic tree construction

To reconstruct the evolutionary history of tumor samples, we employed the BuildTree module in PhylogicNDT. Using the mut_ccf_file generated in the previous step, BuildTree constructed phylogenetic trees that represent the pseudotime position of mutational events.

### Mutational signature analysis

Mutation signatures were inferred utilizing the R package sigminer (accessible at https://github.com/ShixiangWang/sigminer).^58^ The analysis utilized 96 mutation vectors, generated by somatic single nucleotide variants (SNVs) based on six base substitutions (C > A, C > G, C > T, T > A, T > C, and T > G). These vectors were constructed considering 16 possible combinations of neighboring bases for each substitution, serving as input data to determine their contributions to the observed mutations. The sigminer package applied a nonnegative matrix factorization (NMF) approach to analyze the 96 × 113 matrix (representing mutational context-by-sample) in relation to the known COSMIC cancer signatures (available at https://cancer.sanger.ac.uk/cosmic/signatures)^59^ and to infer their exposure contributions. SNP and indels were annotated by mapping to the Catalogue of Somatic Mutations in Cancer (COSMIC). Mutations not described in COSMIC were identified as “unknown”.

### Next-generation sequencing

Next-generation sequencing (NGS) was performed on genomic DNA isolated from frozen specimens using NextSeq platform. We have available matched normal tissue for sequencing. Total RNA was extracted from the samples, and the integrity of the extracted RNA was assessed using agarose gel electrophoresis, while RNA concentration was accurately determined using a NanoDrop. mRNA was isolated from the total RNA using magnetic beads with oligo-dT, and the captured mRNA was then fragmented. Next, the first and second strands of cDNA were synthesized using reverse transcriptase, followed by end repair of the reverse transcription products and the addition of an A base at the 3’ end. The fragment was then ligated with sequencing adapters. The ligated products were purified to remove incomplete ligation products and empty adapter self-ligated products, and then PCR amplification was performed using primers complementary to the adapter sequences. Finally, the sequencing library was purified using magnetic beads. After library construction, the library concentration was measured with Qubit, and the library fragment length was analyzed with an Agilent Fragment Analyzer to ensure library quality. Once the library passed quality control, it was sequenced using the Illumina Novaseq 6000 platform with PE150 sequencing. The average RNA-Seq depth of coverage is 100X.

### Cox regression and survival analysis

Overall survival (OS) distributions were depicted using Kaplan−Meier curves, and statistical significance was assessed through the log-rank test. The association between overall survival and clinical phenotypes was explored through both univariate and multivariate analyses employing the Cox proportional hazards model. In the multivariate analysis, covariates with univariate *P* values < 0.05 were included, and adjustments for stages and lymphatic metastasis were made for prognosis analysis. The R package survival (https://github.com/therneau/survival) facilitated Cox regression and survival analysis.

### Experimental statistical analyses

Data were presented as mean ± SD. Statistical analyses utilized R packages (version 4.2.1, https://cran.r-project.org/)^60^ unless otherwise specified. Student’s t-test, one-way ANOVA, or two-way ANOVA (two-sided) were applied. Statistical significance was considered at *p < 0.05, **p < 0.01, ***p < 0.001, ****p < 0.0001. Adjustment of p-values to the false discovery rate (FDR) was performed using the Benjamini-Hochberg procedure in multiple comparisons.

### Differential gene expression analyses

The DEseq2^61^ package (v1.45.0) in R was employed for differential expression analysis comparing clinical phenotype. Differentially expressed genes were identified using standard cutoffs (*p* < 0.05, |log2(FoldChange)| ≥ 1).^62^ When identifying differentially expressed genes (DEGs) between clinical subgroups (Stage I–II versus Stage III–IVA; LNM versus nLNM), the comparisons were exclusively performed using tumor samples from the respective patient groups.

### GO enrichment analysis

GO enrichment analysis was performed using Metascape.^63^ The protein-coding genes were selected as background. In this study, the Metascape^63^ tool was utilized for separate KEGG^64^ and GO^65^ enrichment analyses of differential genes, with a significance enrichment threshold set at *P* < 0.01.

### EpK score

To construct the EpK gene set, we integrated four keratinization- and epithelium-associated pathways that showed significant enrichment in our analysis: GO:0030855 (epithelial cell differentiation), GO:0008544 (epidermis development), GO:0030216 (keratinocyte differentiation), and GO:0009913 (epidermal cell differentiation), Take the intersection genes of these pathways and delete genes with FPKM mean less than 1 in all samples. The EpK gene set (*n*_genes_ = 78) was then subjected to Gene Set Variation Analysis (GSVA) using the R package GSVA to calculate pathway activity scores across samples. The GSVA algorithm transforms gene-level expression data into pathway-centric scores, enabling quantification of the enrichment level of the EpK gene set in each sample. Samples with GSVA scores >0 were classified as the EpK-high group, reflecting robust activation of keratinocyte differentiation and epidermal development programs. Conversely, samples with scores <0 were assigned to the EpK-low group, indicating suppressed activity of these pathways. This thresholding strategy was based on empirical distributions of GSVA scores and ensured clear separation between biologically distinct subgroups.

### Consensus clustering analysis

Unsupervised clustering of the patient samples at the different molecule levels was performed with the ConsensusClusterPlus R package (v1.70.0).^66^ Samples were clustered using Pearson correlation as the distance measure. The consensus matrix of *k* = 3 showed clear separation among clusters. Taken together, ESCC clusters were defined using *k*-means consensus clustering with *k* = 3. Additionally, an integrated clustering analysis utilizing genomic and transcriptomic data was conducted using the COCA (Cluster-Of-Clusters Analysis) methodology, as implemented in the R package “coca” (https://cran.r-project.org/web/packages/coca/vignettes/coca-vignette.html).^28^

### Estimation of immune cell types from bulk RNA-seq profiles

To describe immune content from bulk RNA-seq samples, we used four central algorithms: CIBERSORT^29^, Robust Partial Correlations^30^, DeconRNASeq^31^ and FARDEEP.^32^ In order to objectively evaluate the abundance of different cells, we took the mean of the results from different methods.

To analyze the cell states and communities within cohort 1, we utilized the online version of EcoTyper (https://ecotyper.stanford.edu/carcinoma/). By inputting transcriptomic profiles from both tumor and adjacent normal tissues, we obtained the corresponding cell community annotations and results.

### Gene-set analysis

Gene set enrichment analysis (GSEA) analysis was performed using clusterProfiler (v4.14.0). GSVA was performed using the GSVA R package (version 1.30.0).^67^ We selected gene sets of curated signaling pathways from the MSigDB Database (v2024.1, https://www.gsea-msigdb.org) to identify pathways enriched in different limb mesenchymal subsets. The gene-by-cell matrix was converted to gene-set-by-cell matrix, and GSVA scores were computed for gene sets with a minimum of 5 detected genes.

### Quantitative PCR (qPCR) detection of S100A8/A9 mRNA

Fresh tumor tissues were homogenized, and total RNA was extracted using the FastPure RNA Extraction Kit (Vazyme, RC112-01). RNA concentration was measured using a NanoDrop spectrophotometer, and cDNA was synthesized via reverse transcription. The cDNA templates were then mixed with primers for S100A8 (Beyotime, QH05433S), S100A9 (Beyotime, QH05437S), and β-actin (Beyotime, QH00001S), along with SYBR Master Mix (Vazyme, Q312-02), and analyzed using a quantitative PCR system for mRNA expression levels.

### ELISA for serum S100A8/A9 detection

Whole blood samples were allowed to clot naturally, followed by centrifugation to collect serum. The S100A8/A9 ELISA kit (CUSABIO, E12149h) was equilibrated to room temperature for 30 min. Standards and serum samples were added to a 96-well plate and incubated at 37 °C for 2 h. After washing, a biotin-conjugated antibody was added and incubated for 1 h, followed by HRP-conjugated streptavidin incubation for another hour. After further washing, chromogenic substrate was added for 15–30 min, and the reaction was stopped. Absorbance at 450 nm was measured using a microplate reader, and sample concentrations were calculated based on the standard curve.

### Immunohistochemistry and multiplex Immunofluorescence

FFPE primary ESCC specimens were used to prepare 4 μm-thick sections. IHC for S100A8/A9 complex was performed according to the manufacturer’s protocol using the S100A8/A9 complex antibody (ab288715, Abcam, 1:5000). To ensure objective comparisons, all tissue specimens were processed and immunostained simultaneously. IHC staining was evaluated using the immunoreactivity score (IRS).^68,69^ Staining intensity was categorized into four levels: no staining (0), weak (1), moderate (2), and strong (3), and was then multiplied by the proportion of positively stained cells: none (0), <10% (1), 10–50% (2), 51–80% (3), and >80% (4). Two independent reviewers, blinded to patient outcomes, assessed the staining results. Discrepancies were resolved through discussion to reach a consensus. The intensity of staining was defined in 4 categories (no staining = 0, weak staining = 1, moderate staining = 2, and strong staining = 3) and multiplied by the proportion of positive cells (no = 0, <10% = 1, 10–50% = 2, 51–80% = 3, >80% = 4). Two independent persons, blinded to the outcomes of patients, reviewed staining images. For cases with different results, a consensus was reached through discussion.

Tumor tissues were embedded in OCT compound and cryosectioned at 6 μm thickness. Sections were fixed with 4% paraformaldehyde for 10–20 min, then washed with PBS and blocked with 5% goat serum for 30 min. Primary S100A8/A9 complex antibody (Abcam, ab288715) was applied and incubated overnight at 4 °C. After PBS washing, corresponding secondary antibodies were incubated for 50 min. Nuclei were counterstained with DAPI, and slides were mounted and examined under a fluorescence microscope. Image analysis was performed using QuPath software.

Normal tissues were embedded in OCT compound and cryosectioned at 6 μm thickness. Sections were fixed with 4% paraformaldehyde for 10–20 min, then washed with PBS and blocked with 5% goat serum for 30 min. Primary antibodies (S100A8, Abclonal-A15315; S100A9, Abclonal-A9842) were incubated overnight at 4 °C. After PBS washing, corresponding secondary antibodies were incubated for 50 min. DAPI was used for nuclear staining. Slides were mounted and visualized using a fluorescence microscope. Image analysis was conducted using QuPath software.

After deparaffinization and antigen retrieval, sections were blocked with 3% BSA for 30 min. The following primary antibodies were sequentially applied and incubated overnight at 4 °C: EpCAM (Abcam, ab223582), HIF-1α (Abcam, ab279654), Vimentin (Abcam, ab8978), FAP (Abcam, ab314456), α-SMA (CST, 19245), CD3 (Abcam, ab135372), CD4 (Abcam, ab133616), CD8 (Abcam, ab237709), FOXP3 (Abcam, ab20034), and PD-1 (Abcam, ab237728). After each primary antibody incubation, slides were incubated with HRP-conjugated secondary antibodies at room temperature for 30 min, followed by a 5 min incubation with fluorophore-conjugated tyramide signal amplification reagent. Each staining cycle was followed by PBS washing and 100 °C heat-induced antibody stripping. Finally, slides were counterstained with DAPI, and images were acquired using the AKOYA Vectra Polaris system. Quantitative analysis was performed using QuPath.

## Supplementary information

Supplementary materials

Supplementary Table 2

Supplementary Table 3

Supplementary Table 4

Clinical trial protocol

## Data Availability

The raw sequence data reported in this paper have been deposited in the Genome Sequence Archive in National Genomics Data Center, China National Center for Bioinformation/Beijing Institute of Genomics, Chinese Academy of Sciences (DNA accession number: HRA009653, RNA accession number: HRA009717) that are publicly accessible at https://ngdc.cncb.ac.cn/gsa-human.
