# Supplementary material for: Comprehensive genomic and transcriptomic analyses reveal prognostic stratification for esophageal squamous cell carcinoma
Source: Signal Transduct Target Ther. 2025 Jul 17;10:223. doi: 10.1038/s41392-025-02306-8 (PMC12267757; doi:10.1038/s41392-025-02306-8)
Supplement: Supplementary file 1 — Supplementary materials [file 41392_2025_2306_MOESM1_ESM.docx]

Supplementary Materials for

**Comprehensive Genomic and Transcriptomic Analyses Reveal Prognostic Stratification for Esophageal Squamous Cell Carcinoma**

#Jian Gao, #Qiming Wang, #Fangqiu Fu, #Yue Zhao, #Teng Yang, Xiangze Li, Yihua Sun, Hong Hu, Longfei Ma, Longsheng Miao, Xiaoyang Luo, Ting Ye, Yiliang Zhang, Yang Zhang, Ziling Huang, Hang Li, Longlong Shao, Midie Xu, Kuaile Zhao, Shiyue Zhang, Mou Zhang, Jun Wang, Chong Dai, Xiaoxiao Shang, Tingyi An, Yawei Zhang, Jiaqing Xiang, Zhiwei Cao*, Bin Li* and Haiquan Chen*

#Authors contributed equally to this work

Correspondence to: zwcao@fudan.edu.cn, lb0256327@hotmail.com, hqchen1@yahoo.com

**This PDF file includes:**

Figures. S1 to S7

Tables S1

**Other Supplementary Materials for this manuscript include the following:**

Three additional supplementary tables (Supplementary Tables 2–4) are provided as separate files due to their detailed content.

Table S2: Top 3000 genes in the Somatic Mutations Matrix

Table S3: Expression Matrix (FPKM) of Top 20 High Mutation Genes

Table S4: Differences in gene mutations of FU-ESCC Subtypes

Figure. S1.


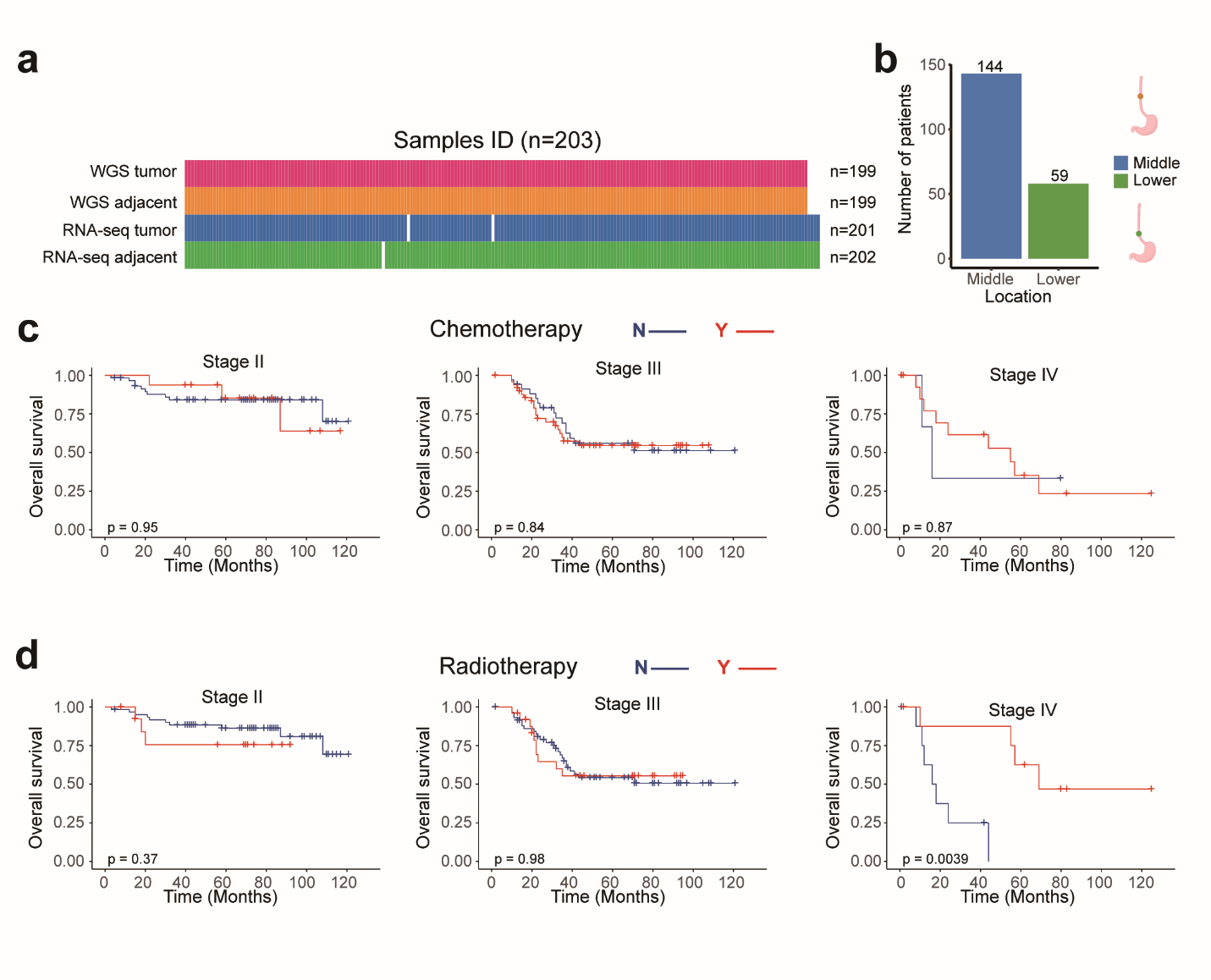


**Supplementary Figure 1.** Supplemental information for the Cohort 1. **(a)** Sequencing data for each patient in Cohort 1. Filled squares indicate the sequencing data contributed by the patient for this study, while blank squares represent insufficient tissue samples for sequencing. **(b)** Tumor location. **(c-d)** The impact of chemotherapy or radiotherapy on OS across different stages in Cohort 1. Stage I patients are excluded, as neither radiotherapy nor chemotherapy is recommended.

Figure. S2.


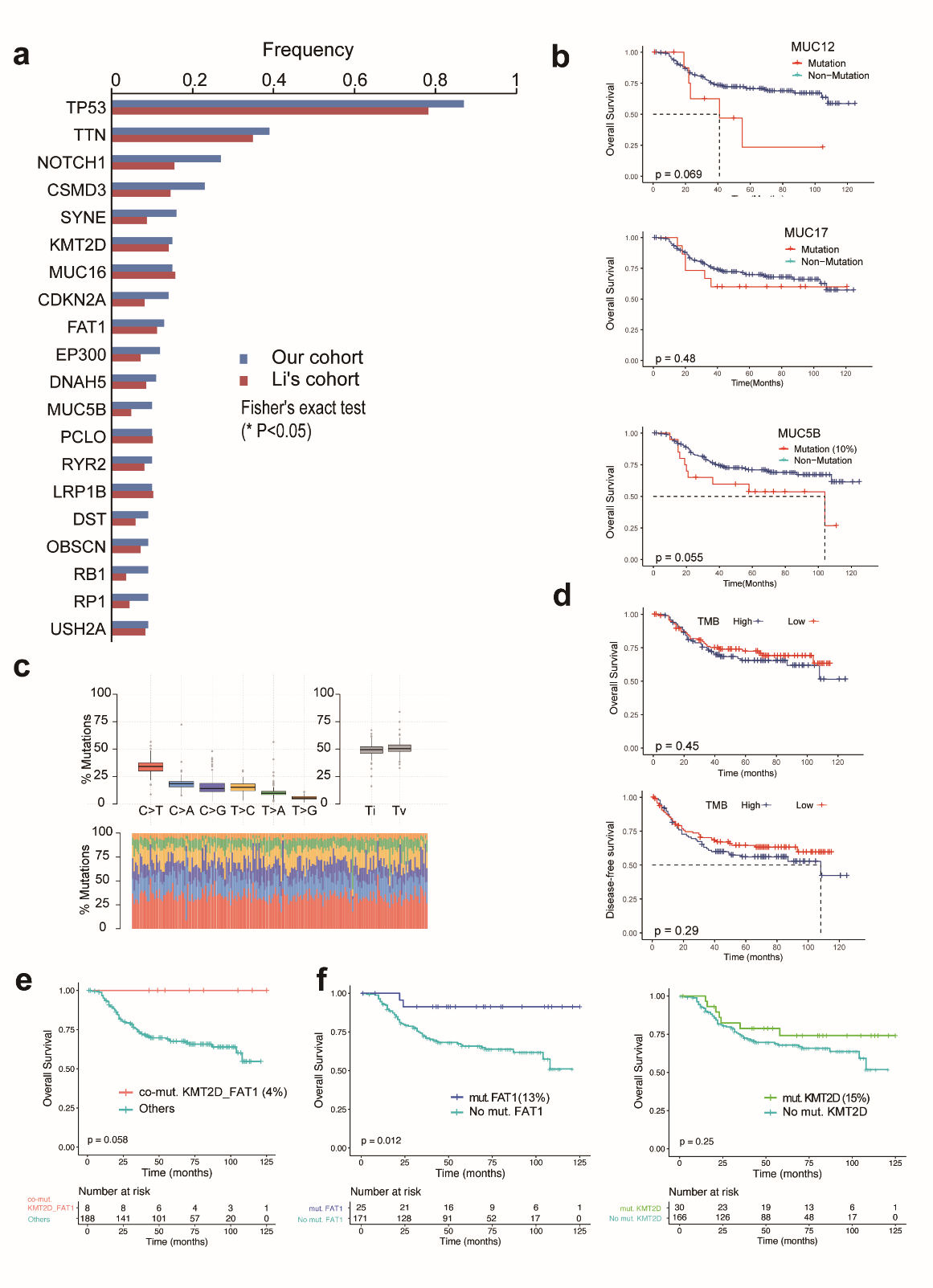


**Supplementary Figure 2.** Associations between mutations and prognosis. **(a)** Comparison of high-frequency mutations identified in this study with those reported in previous studies. **(b)** Association between individual gene mutations and OS. **(c)** Transition (Ti) and transversion (Tv) mutations in cohort1. **(d)** Association between TMB levels and OS. **(e-f)** Association between co-mutated gene pairs and OS.

Figure. S3.


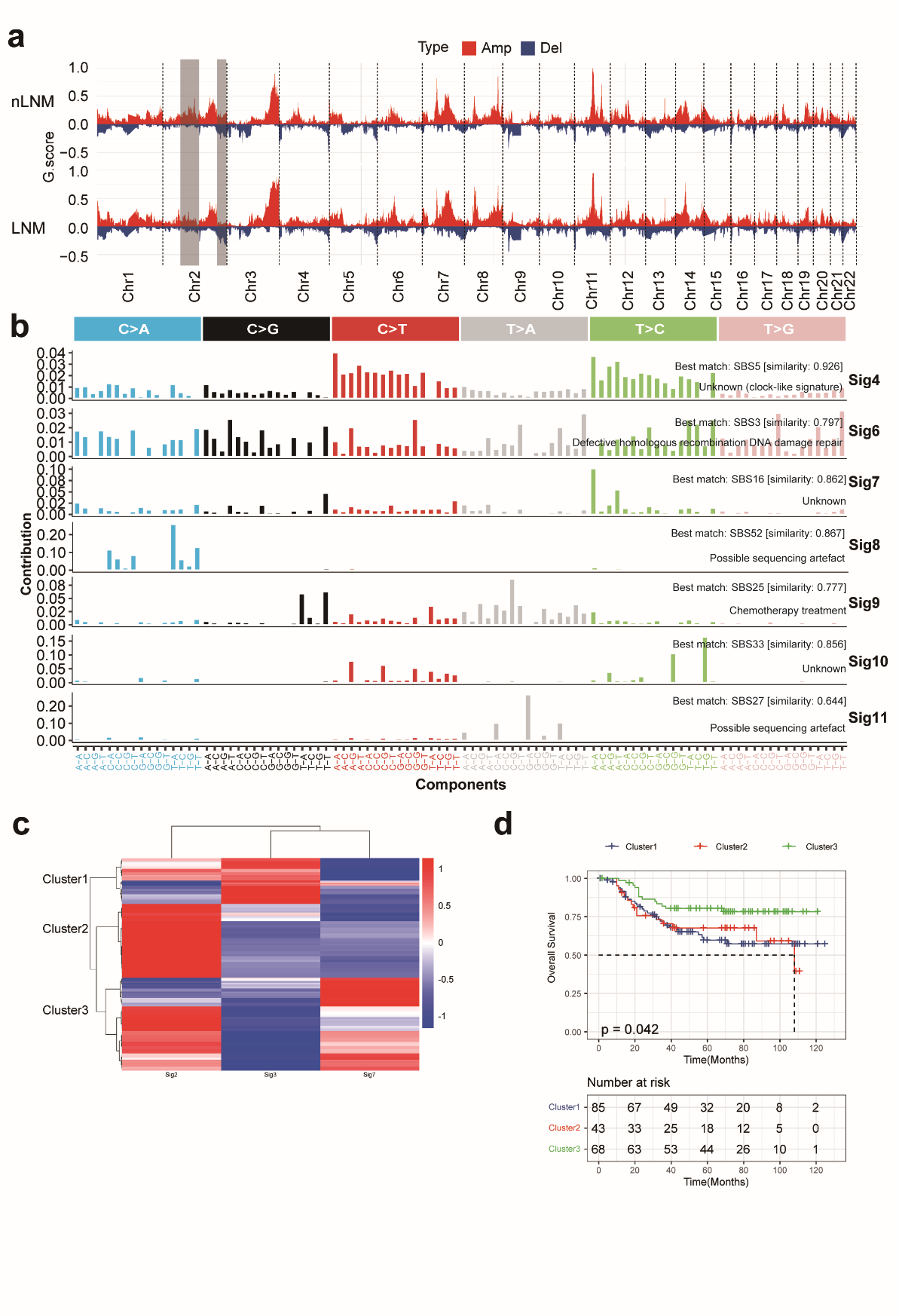


**Supplementary Figure 3.** CNVs, mutational signatures, and gene expression patterns. **(a)** Comparison of copy number variations (CNVs) between nLNM and LNM groups. **(b)** Mutational signatures showing weak correlation (Pearson r < 0.8) or unclear correspondence with known COSMIC signatures. **(c)** Patient clustering based on the relative contribution of mutational signatures (sig2, sig3, sig7). **(d)** Kaplan–Meier analysis of survival differences among mutation-signature clusters.

Figure. S4.


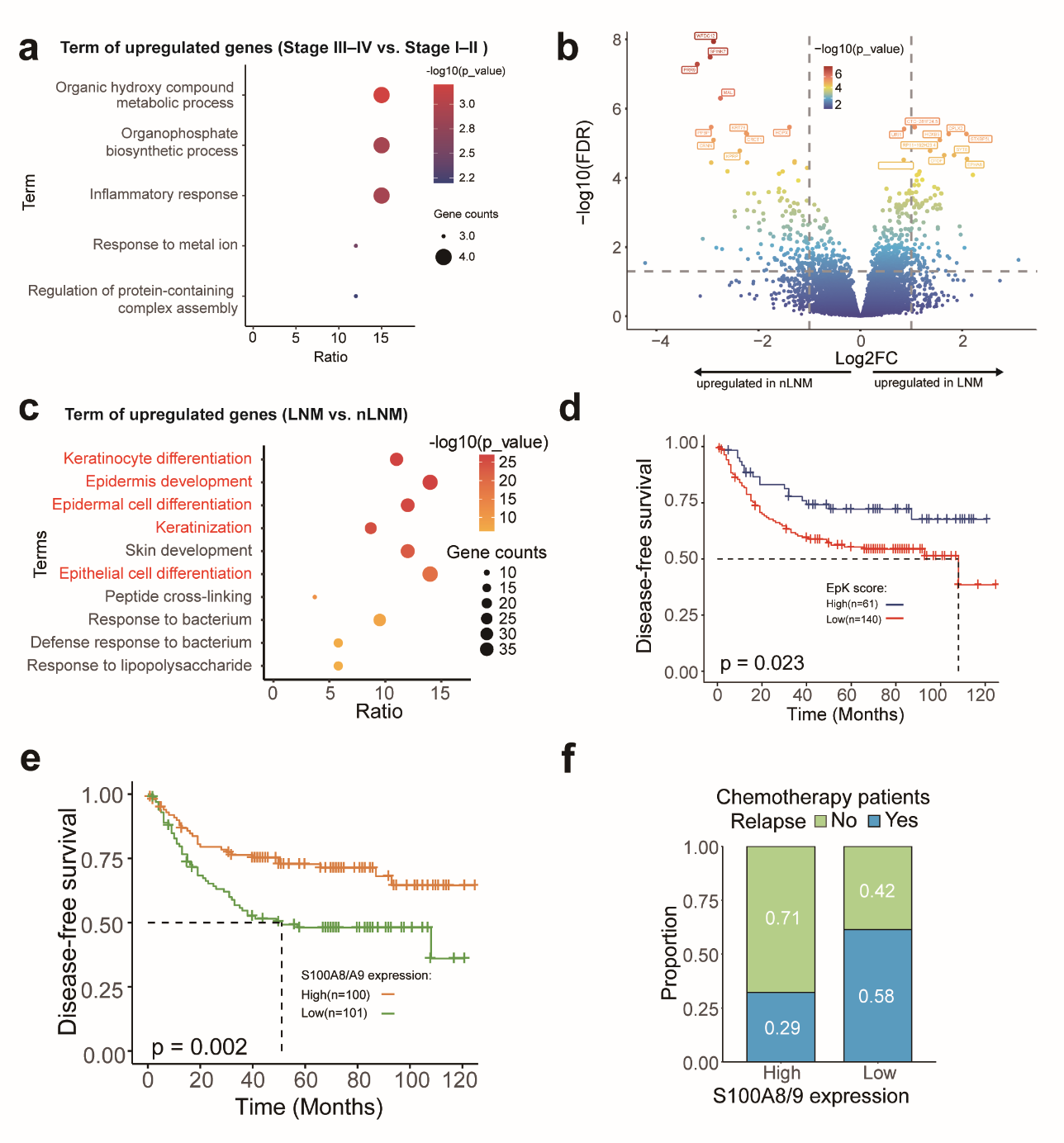


**Supplementary Figure 4.** Gene and functional analyses across different clinical groups. **(a)** GO enrichment analysis of upregulated differentially expressed genes (DEGs) in Stage III–IV compared to Stage I–II patients. **(b)** DEGs identified between LNM and nLNM groups. **(c)** GO enrichment analysis of DEGs in nLNM versus LNM groups. **(d)** Kaplan–Meier analysis of DFS based on the EpK score in ESCC patients. **(e)** Kaplan–Meier analysis of DFS in patients stratified by high versus low expression of the S100A8/A9 gene expression (median as cutoff). **(f)** Post-chemotherapy recurrence rates in patients (n=82) with high versus low S100A8/A9 expression levels.

Figure. S5.


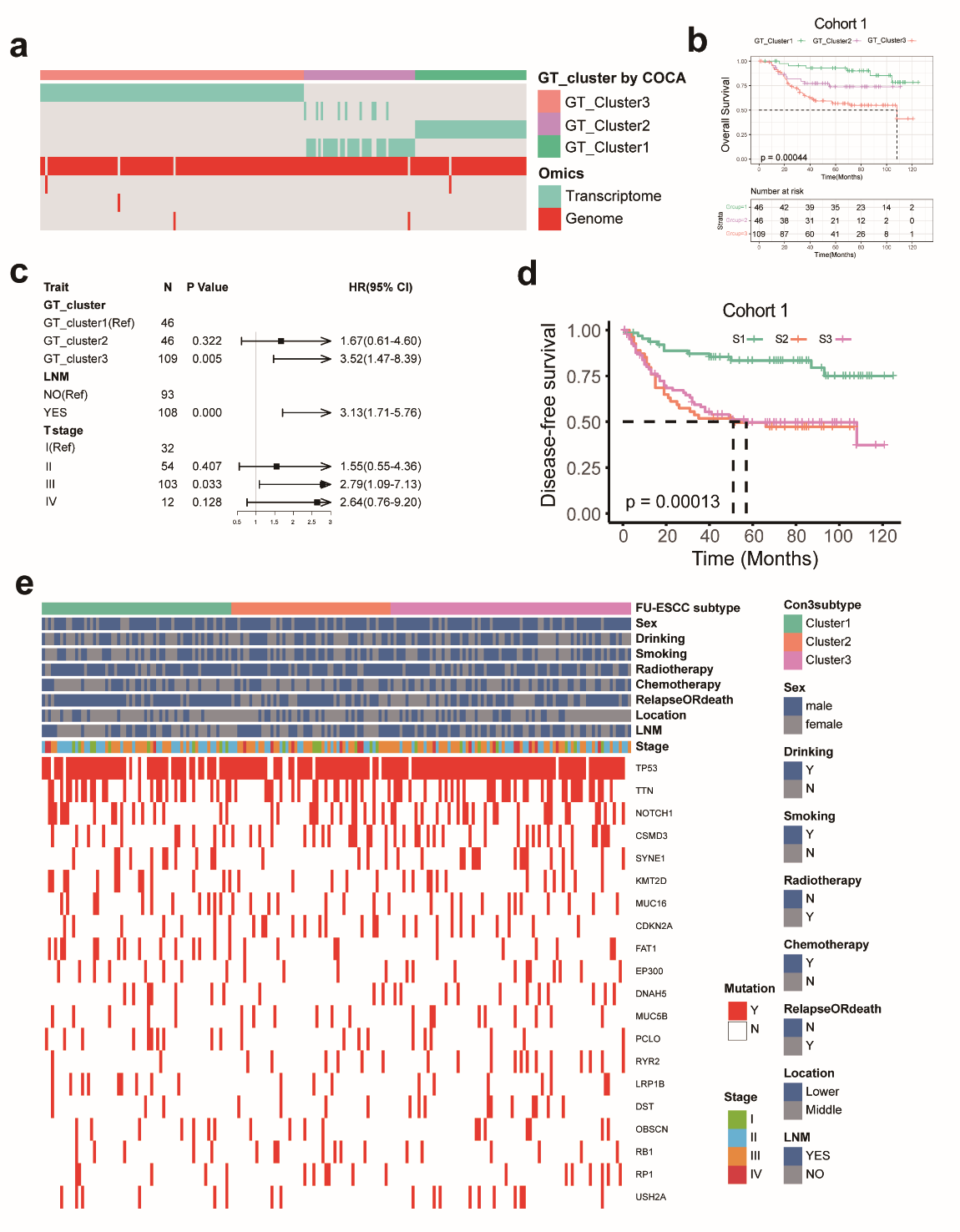


**Supplementary Figure 5.** Genomic-transcriptomic clustering and FU-ESCC subtyping in Cohort 1. **(a)** Genomic-transcriptomic (GT) clustering based on integrated transcriptomic and genomic features. **(b)** Kaplan–Meier survival analysis of OS across the three GT clusters. **(c)** Multivariate Cox regression analysis incorporating GT clusters, clinical stage, and LNM status. **(d)** Kaplan–Meier analysis of DFS across the three FU-ESCC subtypes. **(e)** Clinical and mutational characteristics associated with each FU-ESCC subtype.

Figure. S6.


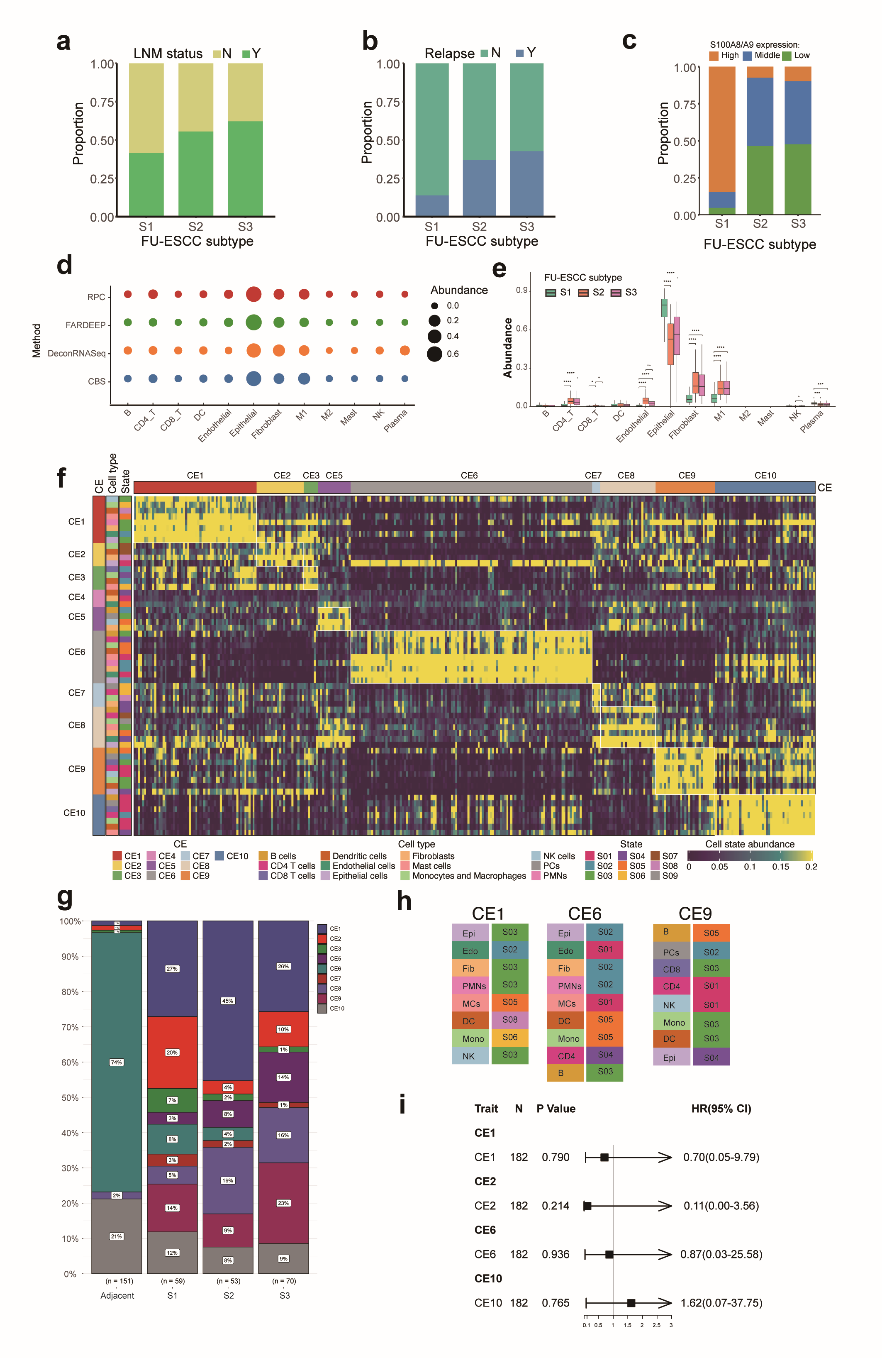


**Supplementary Figure 6.** Clinical characteristics and cellular composition analysis of FU-ESCC subtypes in Cohort 1. **(a–b)** LNM rates and recurrence rates across the subtypes. **(c)** Comparison of S100A8/A9 expression levels among the subtypes. All patients were ranked by expression level and evenly divided into high, medium, and low groups. **(d)** Estimating cell type proportions across subtypes by four deconvolution methods. **(e)** Cellular compositions among the subtypes. Cell type proportions were averaged across the four methods. **(f)** Predicted cell types, cell states, and cell ecotypes using EcoTyper. Transcriptomic data from tumor and adjacent tissues were used as input. The left color bar indicates the ecotype, cell type, and associated state for each cell state. Each column represents one sample; the ecotype with the highest score in each sample was assigned as the dominant CE and was labeled at the top of the column. **(g)** Distribution of cell ecotypes across the three subtypes and adjacent samples. **(h)** Cell types and cell states corresponding to CE1, CE6, and CE9. **(i)** Univariate Cox regression analysis of CE1, CE2, CE6, and CE10.

Figure. S7.


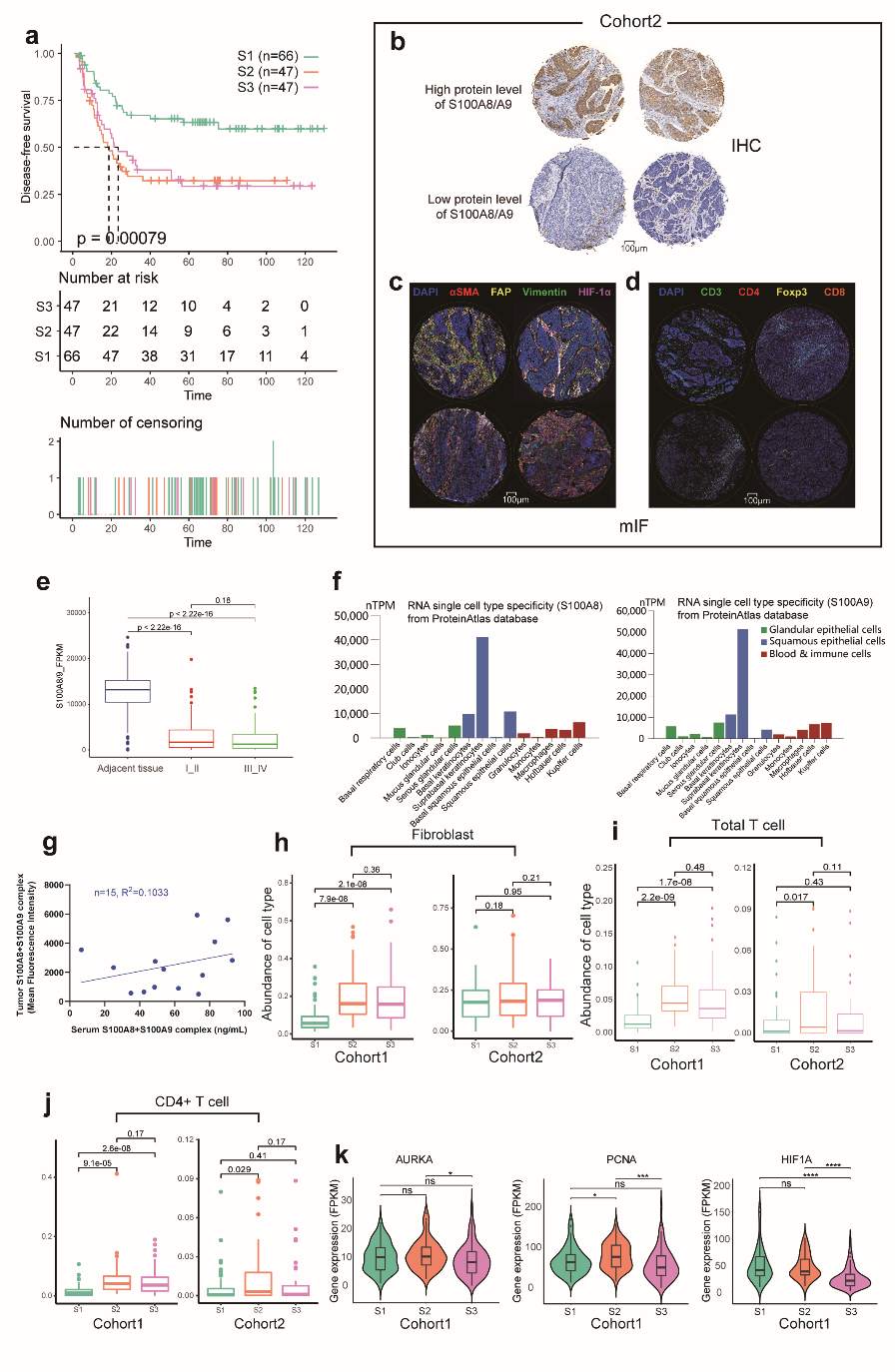


**Supplementary Figure 7.** Validation of FU-ESCC subtype characteristics in Cohort 2. **(a)** Kaplan–Meier analysis of DFS across the three FU-ESCC subtypes. **(b)** Immunohistochemistry staining of S100A8/A9 complex in TMAs. **(c-d)** Representative multiplex immunofluorescence images of TMAs stained for α-SMA, Vimentin, FAP, and HIF-1α; CD3, CD4, CD8, and Foxp3. **(e)** Expression levels of S100A8/A9 in Cohort 1. **(f)** Expression of S100A8 and S100A9 across healthy cell types based on The Human Protein Atlas database. **(g)** Linear regression analysis assessing the correlation between serum and tumor levels of the S100A8/A9 complex (n = 15; samples independent of Cohort 1 and 2; based on frozen tissue sections). **(h–j)** Comparison of cellular compositions across two cohorts. In cohort2, Fibroblast were identified as α-SMA+; Total T cell were identified as CD3+; CD4+ T cell were identified as CD3+ and CD4+. **(k)** Comparison of proliferation-associated gene markers across FU-ESCC subtypes.

Table S1.

|  | |
| --- | --- |
| Variables (n=203) | |
| Age (years), median (IQR) | 62(57,67) |
| Sex, n (%) | |
| Female | 42(20.7) |
| Male | 161(79.3) |
| Alcohol, n (%) | |
| Ever | 102(50.2) |
| Never | 101(49.8) |
| BMI, mean±SD | 23.9±2.8 |
| Tumor location, n (%) | |
| Middle | 144(70.9) |
| Lower | 59(29.1) |
| pT, n (%) | |
| T1 | 32(15.8) |
| T2 | 54(26.6) |
| T3 | 105(51.7) |
| T4 | 12(5.9) |
| pN, n (%) | |
| N0 | 95(46.8) |
| N1 | 59(29.1) |
| N2 | 34(16.7) |
| N3 | 15(7.4) |

**Supplementary Table 1.** Patient Characteristics of enrolled patients
