# Supplementary material for: Comprehensive genomic and transcriptomic analyses reveal prognostic stratification for esophageal squamous cell carcinoma
Source: Signal Transduct Target Ther. 2025 Jul 17;10:223. doi: 10.1038/s41392-025-02306-8 (PMC12267757; doi:10.1038/s41392-025-02306-8)
Supplement: Supplementary file 5 — Clinical trial protocol [file 41392_2025_2306_MOESM5_ESM.docx]

**OFFICIAL TITAL**

Esophagectomy: Three-field lymphadenectomy versus. two-field lymphadenectomy for thoracic middle and lower esophageal cancer

**SETTING**

Fudan University Shanghai Cancer Center

**RESPONSIBLE PARTY**

Haiquan Chen MD.

*This study was discussed in the Multidisciplinary Treatment Team for Thoracic cancer, approved by the Institutional Review Board of Fudan University Shanghai Cancer Center, and registered in Clinicaltrial.Gov under number NCT01807936.*

1. **Background:**

Esophageal carcinoma is an aggressive disease with a poor prognosis. Surgical resection with radical lymphadenectomy remains the basic method of management of this malignancy. Lymph node metastasis is one of the important factors in predicting the prognosis of patients with esophageal carcinoma, but the extent of lymph node dissection is still in debate. In the randomized trial of transhiatal compared with transthoracic esophagectomy for adenocarcinoma from the Netherlands, patients with positive lymph nodes potentially benefited from transthoracic esophagectomy with radical lymphadenectomy) (Hulscher JB, N Engl J Med. 2002). However, cervical LNM are common in esophageal cancer, with the incidence more than 20%. To improve the survival, three-field lymphadenectomy was developed to remove all potentially positive lymph nodes in the neck, mediastinum and upper abdomen. However, the value of three-field lymphadenectomy remains controversial due to the lack of prospective evidence. To date, only 2 randomized trials compared three-field lymphadenectomy and two-field lymphadenectomy, but the results are conflicting and the studies underpowered (Kato H, Ann. Thorac. Surg. 1991; Nishihira T, Am. J. Surg. 1998.)

1. **Purpose:**

The purpose of this study is to test two different extents of lymphadenectomy (Cervical-thoracic-upper abdominal three-field lymphadenectomy and thoracic-upper abdominal two -field lymphadenectomy) for patients with thoracic middle and lower esophageal cancer. This research is being done to see whether one extent of lymphadenectomy is superior than the other with better long-term outcome and acceptable postoperative short-term outcome or not.

1. **Trial:**
2. Allocation: Randomized (sealed envelope method).
3. Endpoint Classification: Efficacy Study.
4. Intervention Model: Parallel Assignment.
5. Masking: Open Label.
6. Primary Purpose: Treatment.
7. Single-institutional.
8. **Study protocol:**


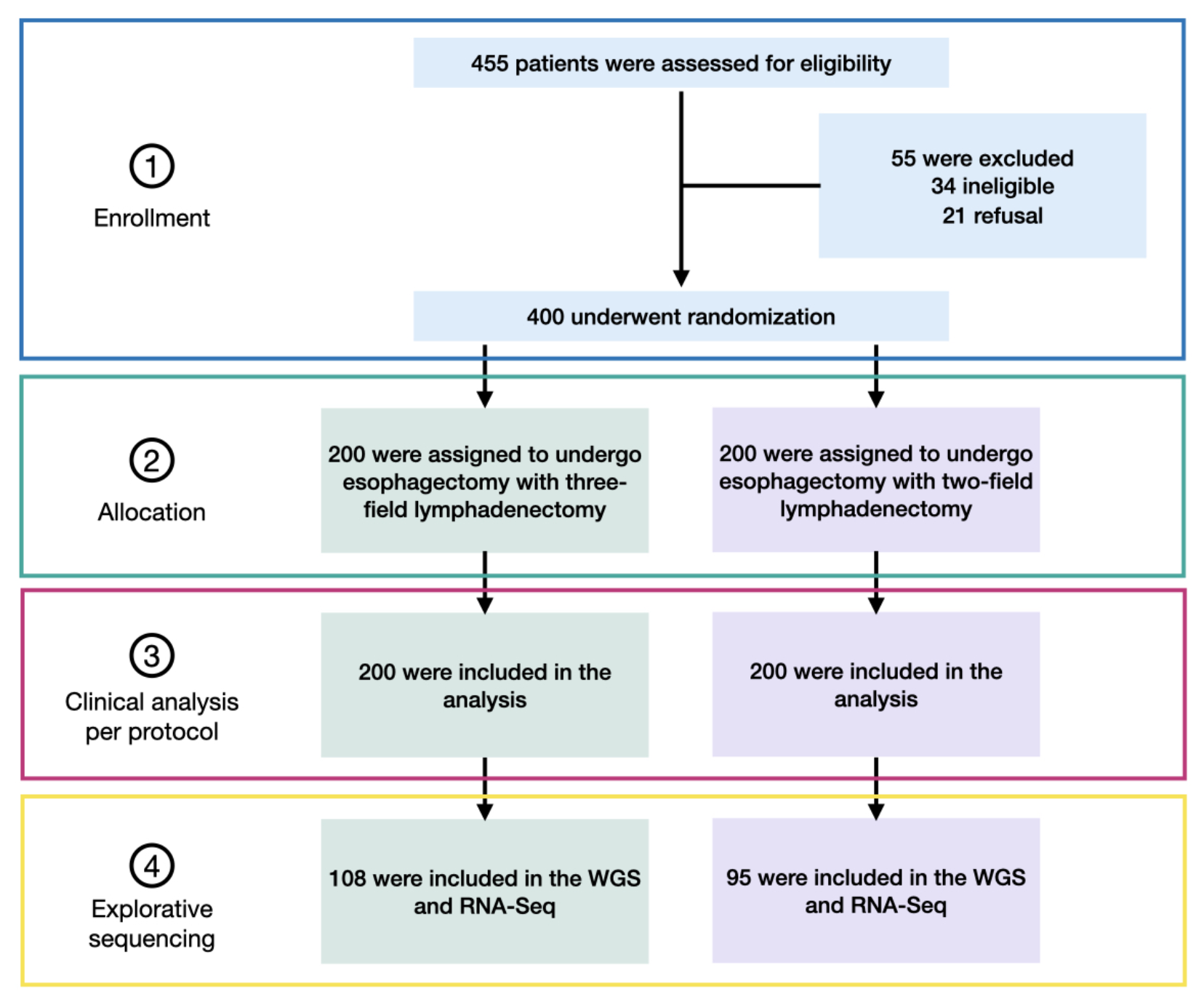


Figure 1. Screening, randomization, follow-up and explorative sequencing of the patients.

**4.1 Estimated Enrollment:** 400.

PASS (power analysis and sample size software) were used for sample size calculation. Previous data indicated a 15% difference in 5-year survival between two-field lymphadenectomy (30%) and three-field lymphadenectomy (45%). With a three-year accrual and an estimation of 10% loss of follow-up, 200 patients per study arm were necessary, using 80% statistic power.

**4.2 Randomization:**

Randomization, by the sealed envelope method, took place on the morning of planed surgery day. Sealed envelopes were prepared and provided by the Department of Biostatistics, Fudan University.

**4.3 Study Start Date:** March 2013

1. **Study Completion Date:** December 2019
2. **Primary Completion Date:** November 2016

**4.4 Surgery and postoperative treatment.**

Surgery was performed by consultant thoracic surgeons who had performed at least 50 esophagectomies each year. Esophageal resection specimens were histopathologically assessed by experienced pathologists using a standardized protocol in which site and size of the primary cancer, sample margins and tumor differentiation were recorded in addition to presence of lymphovascular invasion. All lymph nodes resected were labeled for pathologic examination according to the anatomical sites.

**Three-field lymphadenectomy:**

The patient is placed supine initially. Through an upper midline abdominal incision, gastric tubulization is completed and a feeding jejunostomy is performed. Meanwhile, cervical lymphadenectomy is performed through a collar incision using the technique reported before (Yajima S, Int. J. Surg. Oncol. 2012). Next, the patient is positioned in left lateral decubitus and a right thoracotomy with a muscle-sparing incision is made in the fourth intercostal space. After ligating and dissecting the azygos vein, the esophagus is resected. Subsequently, the gastric tube is delivered into the thorax and a circular stapled end-to-side esophagogastric anastomosis is fashioned in the upper mediastinum. In addition, a nasogastric tube is also positioned in the gastric tube to prevent vomiting and acute gastric tube distension.

Following lymph nodes are resected during cervical lymphadenectomy: Cervical paraesophageal lymph nodes, supraclavicular nodes (no. 104).

Following lymph nodes are resected during mediastinal lymphadenectomy: Upper paraesophageal nodes, nodes along the bilateral recurrent nerves, subcarinal nodes, middle paraesophageal nodes, bilateral hilar lymph nodes, lower paraesophageal nodes, posterior mediastinal lymph nodes, and diaphragmatic nodes.

Following lymph nodes are resected during upper abdominal lymphadenectomy: Paracardial nodes, lesser curvature nodes, greater curvature nodes, left gastric, common hepatic nodes and celiac lymph nodes.

**Two-field lymphadenectomy:**

The patient is placed supine initially. Through an upper midline abdominal incision, gastric tubulization is completed and a feeding jejunostomy is performed. Next, the patient is positioned in left lateral decubitus and a right thoracotomy with a muscle-sparing incision is made in the fourth intercostal space. After ligating and dissecting the azygos vein, the esophagus is resected. Subsequently, the gastric tube is delivered into the thorax and a circular stapled end-to-side esophagogastric anastomosis is fashioned in the upper mediastinum. In addition, a nasogastric tube is also positioned in the gastric tube to prevent vomiting and acute gastric tube distension.

Following lymph nodes are resected during mediastinal lymphadenectomy: Upper paraesophageal nodes, nodes along the bilateral recurrent nerves, subcarinal nodes, middle paraesophageal nodes, bilateral hilar lymph nodes, lower paraesophageal nodes, posterior mediastinal lymph nodes, and diaphragmatic nodes.

Following lymph nodes are resected during upper abdominal lymphadenectomy: Paracardial nodes, lesser curvature nodes, greater curvature nodes, left gastric, common hepatic nodes and celiac lymph nodes.

**Postoperative treatment:**

Patients in both groups received similar postoperative treatment. Patients were extubated at the end of the procedure if physiologically stable, and were then admitted to the intensive care unit and discharged the next day to a thoracic surgery ward. In the first 3 days after surgery, patient-controlled epidural analgesia was the main postoperative pain control system. On postoperative day (POD) 1, patients were encouraged to move out of bed, and enteral nutrition was commenced via the feeding tube. Contrast swallow, not routinely but optionally, was performed on POD 5 or 6. Patients were started on sips of clear liquids on POD 6 and soft solid foods on POD 7, and discharged routinely on POD 7 or 8.

**4.5 Patients.**

**Ages Eligible for Study:** 18 Years to 75 Years

**Genders Eligible for Study:** Both.

**Healthy Volunteers:** No.

**Preoperative evaluation:**

Oncological evaluation included upper GI endoscopy with histologic examination, upper GI barium swallow, computerized tomography of the chest and upper abdomen, and ultrasound of the cervical region. Pulmonary and cardiac functions were also performed to assess the medical operability.

***Inclusion Criteria:***

1. Patients with histologically proven esophageal cancer
2. Patients with cT1-T3/N0-N1 mid or distal third (inferior to carina and 3cm superior to cardia ) operable esophageal lesion. Staging investigations including esophagogastroscopy, chest and abdominal CT scan, barium swallow and selective endoscopic ultrasonography showing no evidence of invading adjacent structure such as spine, bronchus, pericardium, descending aorta and without enlarged cervical and celiac nodes (diameter of short axis greater than 1.5cm) measured at CT scans.
3. Karnofsky performance status greater than or equal to 80%
4. Pulmonary and cardiac function must be acceptable for surgery according to institutional standards.
5. Acceptable hepatic, renal and bone marrow function

***Exclusion Criteria:***

1. Patients with low performance status (Karnofsky score <80%)
2. Past history of malignancy
3. Stage investigations indicating unresectable advanced disease (T4 or M1a,M1b)
4. Patients with any other serious underlying medical condition that would impair the ability of the patient to receive or comply with protocol treatment
5. Chemo and/or radiotherapy before surgery
6. Patients medically unfit for surgical resection
   1. **Data collection**

All collected data will be entered into a statistical software package for subsequent analysis.

- 1. **Postoperative treatment and follow-up**

Postoperative adjuvant treatment was given according to the postoperative pathological findings:

1. Radio-therapy was performed for patients with T3/4 tumors, or positive resection margins.
2. Chemo-therapy was performed for patients with positive lymph nodes.

Patients will be followed up every three months for the first two years and every six months for the third to fifth years and annually thereafter. A detailed history and clinical examination and CT scan, barium swallow and ultrasound will be done routinely on every follow up. Patients were seen either at our outpatient’s clinic or by telephone interview.

**5. Outcomes and measures:**

**5.1 Primary Outcome Measures:**

1. Overall survival: Time form randomization to the death due to any cause.

**5.2 Secondary Outcome Measures:**

1. Disease free survival.

Time from randomization to the first recurrence (locoregional, hematogenous, and others) or death from any cause.

1. Postoperative morbidity and mortality.

Hospital death defined as any death during hospital stay.

Postoperative complications included:

1. Anastomotic leak: Identified clinically or radio-graphically,
2. Respiratory complications: Clinical manifestation of pneumonia or bronchopneumonia confirmed by CT scan
3. Cardiovascular complications: Persistent arrhythmia requiring medical treatment
4. Chylothorax: Appearance of milky fluid from the thoracic drains after onset of enteral nutrition
5. Wound infections: Wound opened and daily bedside dressing is needed.
6. Delayed gastric emptying: Delayed oral food intake and confirmed by contrast swallow.
7. Pleural effusion: A thoracentesis was need.
8. Recurrent nerve injury: Hard to define, and recorded according to the change compared with the voice before surgery identified by patient and surgeon in charge.
9. Locoregional recurrence and recurrence pattern.

Recurrence were classified as locoregional (at the site of the primary tumor, the anastomotic site, or the lymph node), hematogenous, and other.

1. **Statistical analysis plan**

**Primary outcome**

**Overall survival**, calculated from the time of randomization to death from any cause or the time to the last follow-up visit. Overall survival will be compared 3 years after the recruitment.

**Secondary outcomes**

1. **Disease-free survival**，calculated from the time of randomization to date of first recurrence or death from any cause. Disease-free survival will be compared 3 years after the recruitment.
2. Perioperative morbidity and mortality, and oncologic efficacy will be compared when the last patient discharged.
3. Locoregional recurrence and recurrence pattern will be compared 3 years after the recruitment.

All data analyses will be performed with the statistical package SPSS. A *P* value of <0.05 is considered statistically significant.

- 1. Chi-square or Fisher’s exact test will be used to compare categorical data.
  2. Student’s t-test or the Mann-Whitney U test will be used for continuous data.
  3. The Kaplan-Meier method will be used to estimate survival, and the log-rand test will be used to determine significance.
  4. Multivariate Cox regression analysis will be used to identify the risk factors related to overall survival. Multivariate Logistic regression analysis will be used to identify the risk factors related to cervical lymph nodes metastasis.
